# Thirty years of resistance: Zig-zag through the plant immune system

**DOI:** 10.1093/plcell/koac041

**Published:** 2022-02-15

**Authors:** Bruno Pok Man Ngou, Pingtao Ding, Jonathan D G Jones

**Affiliations:** The Sainsbury Laboratory, University of East Anglia, Norwich NR4 7UH, UK; RIKEN Center for Sustainable Resource Science, Yokohama, Japan; The Sainsbury Laboratory, University of East Anglia, Norwich NR4 7UH, UK; Institute of Biology Leiden, Leiden University, Leiden 2333 BE, The Netherlands; The Sainsbury Laboratory, University of East Anglia, Norwich NR4 7UH, UK

## Abstract

Understanding the plant immune system is crucial for using genetics to protect crops from diseases. Plants resist pathogens via a two-tiered innate immune detection-and-response system. The first plant *Resistance (R)* gene was cloned in 1992 . Since then, many cell-surface pattern recognition receptors (PRRs) have been identified, and *R* genes that encode intracellular nucleotide-binding leucine-rich repeat receptors (NLRs) have been cloned. Here, we provide a list of characterized PRRs and NLRs. In addition to immune receptors, many components of immune signaling networks were discovered over the last 30 years. We review the signaling pathways, physiological responses, and molecular regulation of both PRR- and NLR-mediated immunity. Recent studies have reinforced the importance of interactions between the two immune systems. We provide an overview of interactions between PRR- and NLR-mediated immunity, highlighting challenges and perspectives for future research.

## Teaser

A review of major research advances in plant immunity during the last three decades and individual characterized immune receptors, their immune signaling pathways, and interactions between immune systems

## The plant immune system

Plants are constantly challenged by diverse organisms, including viruses, bacteria, fungi, oomycetes, herbivores, and parasitic plants. Disease ensues when a plant is susceptible to any of these organisms. Plants carry powerful defense mechanisms. To cause disease, pathogens usually need to evade detection by the host and/or to suppress these immune responses. Cell-surface pattern recognition receptors (PRRs) in plants recognize conserved pathogen-/damage-/microbe-/herbivore-associated molecular patterns (PAMPs/DAMPs/MAMPs/HAMPs) and activate pattern-triggered immunity (PTI), which restricts pathogenicity. PRRs are plasma membrane (PM)-associated and are usually either receptor-like kinases (RLKs) or receptor-like proteins (RLPs) that lack a protein kinase domain. Pathogens have evolved to evade or suppress PTI through secreted effector molecules, which results in effector-triggered susceptibility (ETS). Plants, in turn, have evolved intracellular nucleotide-binding leucine-rich repeat receptors (NLRs) to detect effectors, which are often encoded by *Resistance (R)* genes, and activate effector-triggered immunity (ETI) upon effector perception. Pathogens might then evolve or diversify or lose effectors to suppress or evade ETI. The interaction between PTI, ETS, and ETI was incorporated into the widely cited “zig-zag-zig” intellectual framework (Jones and Dangl, 2006).

## The alphabet soup digested: nomenclatures applied to the plant immune system

**Figure 1:**
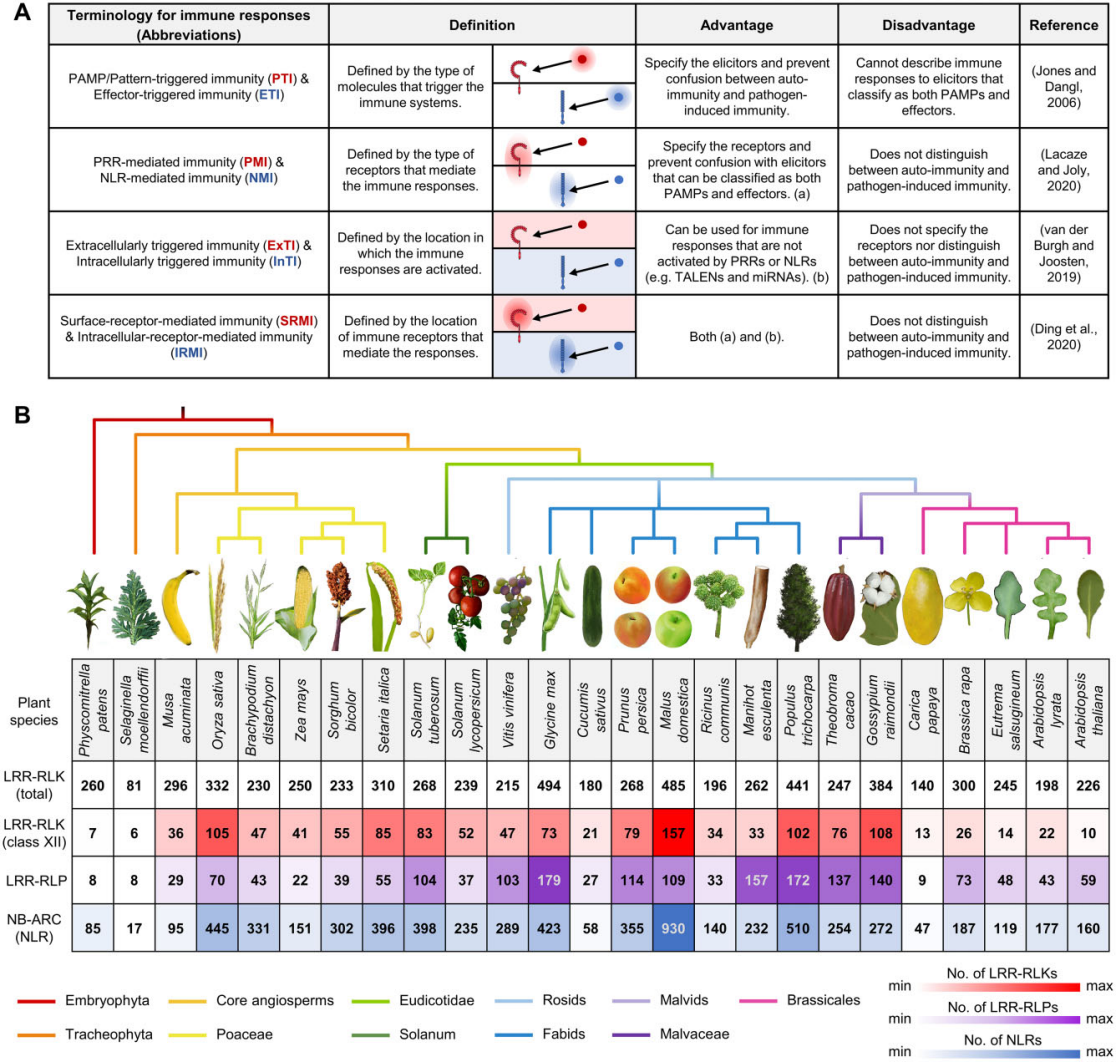
Nomenclatures in plant immunity and the evolution of plant immune receptors. A, Terminology for plant immune responses. Tabular summary of the different terms used to describe plant immune responses. Definitions, advantages, and disadvantages for each of these are included. B, Number of LRR–RLKs, LRR–RLPs, and NLRs in different plant species. Phylogenetic tree illustrating different plant species with the corresponding numbers of LRR–RLKs, LRR–RLK XII (class or subgroup XII), LRR–RLPs, and NLRs. Red heatmap indicates the number of LRR–RLK XIIs, purple heatmap indicates the number of LRR–RLPs, and blue heatmap indicates the number of NLRs. The phylogenetic tree was generated using phyloT (https://phylot.biobyte.de/) based on the NCBI taxonomy database and visualized by iTOL (https://itol.embl.de/). LRR–RLK data were obtained from Dufayard et al. (2017), LRR–RLP data were obtained from Ngou et al. (2022), and NLR data were obtained from Baggs et al. (2020).

PTI was originally an abbreviation for “PAMP-triggered immunity”, mediated by PRRs such as *Arabidopsis thaliana* Flagellin-Sensing 2 (FLS2). ETI is an acronym for “effector-triggered immunity,” which is mostly mediated by NLRs (Jones and Dangl, 2006), but can also involve RLP-mediated detection of apoplastic effectors (Jones et al., 1994). While the terms PTI and ETI are frequently used in the literature, there are limitations to their use in describing specific immune responses (Thomma et al., 2011). For example, the apoplastic effector Avr4 from the tomato (*Solanum lycopersicum*) leaf mold pathogen *Cladosporium fulvum* binds to fungal chitin to retard cell wall degradation by host chitinases and thus the release of *N*-acetyl glucosamine oligomers that activate defense (Joosten et al., 1994; van den Burg et al., 2006). Avr4 is recognized by the tomato cell-surface RLP Cf-4 (Thomas et al., 1997). Thus, while immunity activated by some PRRs can be classified as PTI, others can be classified as ETI, since cell-surface receptors can recognize both PAMPs and apoplastic effectors (Thomma et al., 2011). Other terms have been introduced to classify immune responses based on receptors, such as PRR-mediated immunity and NLR-mediated immunity (Lacaze and Joly, 2020). Immune responses are best defined by the location of recognition by the initiating protein, such as extracellularly triggered immunity and intracellularly triggered immunity (van der Burgh and Joosten, 2019), or surface-receptor-mediated immunity and intracellular-receptor-mediated immunity (van der Burgh and Joosten, 2019; Ding et al., 2020). Each of these terms has its own advantages and should be used with caution (Figure 1A). In this review, we try to minimize the overuse of these acronyms and emphasize immune responses triggered by the corresponding receptors.

## Structural and evolutionary overview of PRR proteins

Plant PRR proteins are either RLPs or RLKs. RLKs consist of an extracellular domain, a transmembrane domain, and cytoplasmic kinase domain. RLPs lack a cytoplasmic kinase domain, and both require co-receptors to transduce immune signals. PRRs are localized to the PM via a transmembrane α-helix or a glycophosphatidylinositol (GPI) anchor (Boutrot and Zipfel, 2017). Both RLPs and RLKs perceive ligands via a range of extracellular domains. These include leucine-rich repeat (LRR), lectin, malectin, lysin motif (LysM), and epidermal growth factor-like domains (Boutrot and Zipfel, 2017).

RLKs are found in *Plasmodium*, plants, and animals but not fungi (Shiu and Bleecker, 2003). Conceivably, RLKs were present in the common ancestors of these organisms but were later lost in the fungi. Plant RLKs underwent remarkable expansion and constitute 60% of the kinases in the Arabidopsis genome (Shiu and Bleecker, 2003). Arabidopsis RLKs can be classified into 44 subfamilies based on their kinase domains (Shiu and Bleecker, 2003). The LRR–RLKs represent the largest subfamily of RLKs and are the best characterized RLKs in plants. A phylogenetic study of 33 plant species concluded that the average number of LRR–RLKs in angiosperms is approximately 250 per species (Dufayard et al., 2017; Figure 1B). LRR–RLKs are further classified into 20 subgroups, with subgroup XII constituting genes involved in pathogen recognition, such as *FLS2*, *EFR*, and *Xa21* (Dufayard et al., 2017). Interestingly, the gene number in the LRR–RLK subgroup XII is highly variable across plant species, indicating that these genes underwent either expansion or contraction in particular lineages (Dufayard et al., 2017; Ngou et al., 2022). Similarly, the LRR–RLPs represent the largest subfamily of RLPs in plants, and the size of this gene family is also highly variable across plant species (Ngou et al., 2022; Figure 1B).

## Structural and evolutionary overview of NLR proteins

NLRs are grouped into three classes according to their N-terminal domains: coiled-coil (CC) NLRs (CNLs), Toll/Interleukin-1 receptor/Resistance (TIR) protein NLRs (TNLs), and RPW8-like CC domain (RPW8) NLRs (RNLs). Both CNLs and RNLs contain N-terminal CC-domains. Plant NLRs carry a nucleotide-binding (NB) domain shared by APAF-1, various plant *R* proteins and CED-4 (together, the NB-ARC domain), and LRR domains at their C-termini. These domains vary between NLRs, and additional noncanonical domains can be integrated into some NLRs (also known as NLR-integrated domains, or NLR-IDs; Sarris et al., 2016). The functions of these domains also vary among NLRs. The LRR domain is involved in direct or indirect recognition of effectors (Krasileva et al., 2010; Ma et al., 2020a; Martin et al., 2020). The NB-ARC domain exhibits ATP binding activity and acts as a switch for NLR activation (Wang et al., 2019b). The CC, TIR, and RPW8 domains function as signaling domains to downstream responses upon NLR activation (Adachi et al., 2019a; Bi et al., 2021; Duxbury et al., 2021; Jacob et al., 2021). Some CC-domains are involved in effector recognition and interact directly with effectors (Avr-Pik) as well as a “guardee” protein (such as RIN4), which is a target of pathogen effectors (Lukasik and Takken, 2009; Kanzaki et al., 2012). The α-helices in both the CC and RPW8 domains were recently shown to form cation channels required for defense signaling (Bi et al., 2021; Jacob et al., 2021). TIR domains can also self-associate or associate with the TIR domains from paired TNLs, which is crucial for their activation (Williams et al., 2014; Duxbury et al., 2020). TIR domains, upon oligomerization, exhibit NADase activity, which leads to the production of variant-cyclic-ADP-ribose (v-cADPR; Horsefield et al., 2019; Wan et al., 2019a). TIR domains also exhibit 2′,3′-cAMP/cGMP synthetase activity (Yu et al., 2021). These small molecules produced by TIR domains likely function in signaling. The ID domain in NLR-IDs functions as a decoy, which enables the NLR to detect effectors targeting proteins with homology to the ID (van der Hoorn and Kamoun, 2008; Sarris et al., 2016; Baggs et al., 2017).

*NLR* genes are present in the genomes of all land plants (Gao et al., 2018). CNLs, TNLs, and RNLs are present in basal angiosperm species such as *Amborella* and *Nymphaea* (Baggs et al., 2020; Liu et al., 2021). However, TNLs are absent from most monocot genomes, indicating that gene loss likely occurred before monocots diverged from dicots (Tarr and Alexander, 2009). The loss of TNLs was also accompanied by the loss of TNL-signaling components, such as ENHANCED DISEASE SUSCEPTIBILITY 1 (EDS1), PHYTOALEXIN DEFICIENT 4 (PAD4), and SENESCENCE-ASSOCIATED GENE 101 (SAG101; Baggs et al., 2020; Liu et al., 2021). The loss of these signaling components may have driven the contraction of TNLs in some angiosperm lineages, or vice versa (Liu et al., 2021). Similar to the LRR–RLK-XII and LRR–RLP, the number of NLRs (or NB-ARC containing proteins) is also highly variable across the angiosperms (Baggs et al., 2020; Liu et al., 2021). Furthermore, the LRR–RLK-XII, LRR–RLP, and NLR gene families have undergone lineage-specific co-expansion or co-contraction (Ngou et al., 2022; Figure 1B). The cause of these concerted expansions and/or contractions is currently unclear but has been proposed to be linked to pathogen pressure and ecological specialization (Plomion et al., 2018; Baggs et al., 2020; Liu et al., 2021; Ngou et al., 2022).

## PRRs involved in pathogen recognition

**Figure 2:**
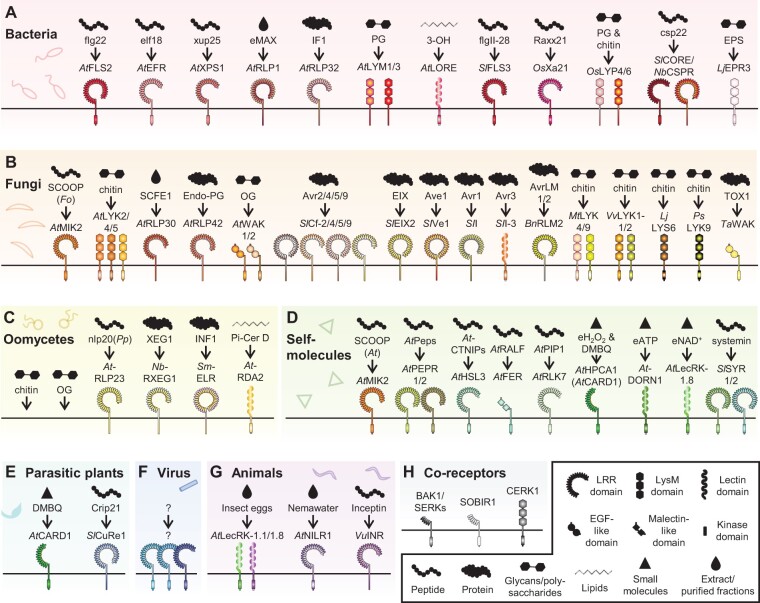
PRRs involved in plant immunity. Characterized PRRs with known elicitors from (A) bacteria, (B) fungi, (C) oomycetes, (D) self-molecules, (E) parasitic plants, (F) viruses, and (G) herbivores. H, PRR co-receptors. Abbreviations for plant species: *A. thaliana*, *At*; *S. lycopersicum*, *Sl*; *O. sativa*, *Os*; *N. benthamiana*, *Nb*; *L. japonicus*, *Lj*; *B. napus*, *Bn; M. truncatula*, *Mt*; *V. vinifera*, *Vv*; *L. japonicus*, *Lj*; *P. sativum*, *Ps*; *T. aestivum*, *Ta*; *S. microdontum*, *Sm*; *P. japonicum*, *Pj*; *V. unguiculata*, *Vu*. Abbreviation for pathogens: *F. oxysporum*, *Fo*; *P. parasitica*, *Pp*. Number of LRR repeats in the LRR–RLKs and LRR–RLPs were predicted by phytoLRR (Chen, 2021). The full name of these PRR genes can be found in Supplemental Data Set 1.

PRRs recognize PAMPs/MAMPs/HAMPs from bacteria, fungi, oomycetes, parasitic plants, and herbivores. Some PRRs also recognize self-molecules, such as DAMPs and other plant endogenous peptides (phytocytokines; Hou et al., 2021). Some PRRs are not involved in direct ligand recognition but function as PRR co-receptors and negative regulators of immune signaling. There are more than 60 characterized immunity-related PRRs with known elicitors, and we attempt here to list those PRRs with known elicitors that are involved in pathogen recognition (Figure 2). Due to space limitations, some PRR gene names are abbreviated: the full gene names can be found in Supplemental Data Set 1.

### PRRs involved in bacterial recognition

Plants perceive a range of PAMPs from bacteria, including peptides, lipids, peptidoglycans (PGs), and polysaccharides. Arabidopsis perceives the bacteria-derived peptides flg22, elf18, and xup25 via the LRR–RLKs *At*FLS2, *At*EFR, and *At*XPS1 and the proteinaceous eMAX and translation initiation factor 1 via the LRR–RLPs *At*RLP1 and *At*RLP32, respectively (Chinchilla et al., 2006; Zipfel et al., 2006; Jehle et al., 2013; Mott et al., 2016; Fan et al., 2021). Other bacterial peptides such as RaxX21, flgII-28, and csp22 are perceived by rice (*Oryza sativa*) *Os*Xa21, tomato *Sl*FLS3, and *Sl*CORE/*Nb*CSPR (from tomato and *Nicotiana benthamiana*), respectively (Pruitt et al., 2015; Hind et al., 2016; Saur et al., 2016; Wang et al., 2016; Luu et al., 2019). The bacterial lipid 3-hydroxydecanoic acid is perceived through the lectin receptor kinase *At*LORE (Kutschera et al., 2019). PGs from bacterial cell walls are perceived by the LysM-containing RLP *At*LYM1/3 and rice *Os*LYP4/6 (Willmann et al., 2011; Liu et al., 2012). Bacterial exopolysaccharides are perceived by the LysM-containing RLK *Lj*EPR3 from *Lotus japonicus* to control rhizobium infections (Kawaharada et al., 2015; Figure 2A).

### PRRs involved in fungal recognition

The fungal cell wall comprises chitin and oligo-galacturonides (OGs), which are perceived by multiple PRRs. Chitin is perceived by LysM-containing RLKs such as *At*LYM2/4/5, *Os*LYP4/6, *Medicago truncatula Mt*LYK4/9, grapevine (*Vitis vinifera*) *Vv*LYK1-1/2, *L.* *japonicus Lj*LYS6, and pea (*Pisum sativum*) *Ps*LYK9 (Wan et al., 2008, 2012; Liu et al., 2012; Faulkner et al., 2013; Cao et al., 2014; Bozsoki et al., 2017; Leppyanen et al., 2017; Brulé et al., 2019). OGs are perceived by the cell wall-associated kinases *At*WAK1/2 (Brutus et al., 2010). *At*WAK1/2 also perceive pectin from the plant cell wall (Kohorn and Kohorn, 2012). The common wheat (*Triticum aestivum*) wall-associated kinase *Ta*WAK perceives the protein SnTox1 from the necrotrophic fungal pathogen *Parastagonospora nodorum* and induces cell death (Shi et al., 2016). In addition to the fungal cell wall, apoplastic effectors from fungal pathogens are recognized by multiple LRR–RLPs. These include *Sl*Cf-2, *Sl*Cf-4, *Sl*Cf-5, *Sl*Cf-9, *Sl*EIX2, *Sl*Ve1, *Sl*Hrc9-4E, *Sl*I, *Sl*I-3, and *Brassica napus Bn*RLM2 (Jones et al., 1994; Dixon et al., 1996, 1998; Thomas et al., 1997; Krüger et al., 2002; Westerink et al., 2004; Rep et al., 2004; Ron and Avni, 2004; Houterman et al., 2008; de Jonge et al., 2012; Larkan et al., 2013; Catanzariti et al., 2015). A proteinaceous elicitor from the fungal pathogen *Sclerotinia sclerotiorum*, sclerotinia culture filtrate elicitor 1, is perceived by *At*RLP30, and fungal endopolygalacturonases (endo-PGs) are perceived by the LRR–RLP *At*RLP42 (Zhang et al., 2013, 2014; Figure 2B).

### PRRs involved in the recognition of oomycetes

The oomycete cell wall is also composed of chitin, endo-PGs, and OGs. Thus, plants also perceive oomycetes via PRRs described in the previous section. In addition, some PRRs recognize specific PAMPs from oomycetes. For example, the glycoside hydrolase XEG1 from *Phytophthora sojae* is recognized by the LRR–RLP *Nb*RXEG1 (Wang et al., 2018d). INF1 elicitin from *Phytophthora infestans* is recognized by the LRR–RLP *Sm*ELR from *Solanum microdontum* (Kamoun et al., 1997; Du et al., 2015b; Domazakis et al., 2020). Arabidopsis *At*RLP23 recognizes a conserved peptide (nlp20) in necrosis and ethylene (ET)-inducing peptide 1-like protein (NLP) from multiple pathogens, including *Phytophthora parasitica* (Böhm et al., 2014; Albert et al., 2015). The Arabidopsis lectin-receptor kinase *At*RDA2 was recently shown to recognize 9-methyl sphingoid base, a PAMP derived from oomycete ceramide (Kato et al., 2021; Figure 2C).

### PRRs involved in self-recognition

Plants perceive DAMPs and phytocytokines from damaged or infected tissues to amplify and modulate immune responses against pathogens. Damage-induced cytosolic calcium influx activates metacaspases, which cleave the DAMP precursor PROPEPs into PEPs (Hander et al., 2019). PEPs are then secreted and perceived by the LRR–RLKs *At*PEPR1/2 (Yamaguchi et al., 2006, 2010). Multiple phytocytokines are upregulated during immunity (Hou et al., 2021). The stress-induced plant signaling peptides CTNIPs are upregulated during PTI and are perceived by the Arabidopsis LRR–RLK *At*HSL3 (Rhodes et al., 2021a). Another defense-induced secreted peptide, PIP1, is recognized by *At*RLK7 (Hou et al., 2014). The Arabidopsis LRR–RLK *At*MIK2 perceives the phytocytokine SCOOP peptides and SCOOP-like peptides from *Fusarium* spp. (Coleman et al., 2021; Rhodes et al., 2021b). Thus, *At*MIK2 is involved in both self and fungal recognition during immunity. Plant PRRs also perceive a range of extracellular (e) self-molecules, such as eH_2_O_2_, eATP, and eNAD. These molecules are perceived by *At*HPCA1 (also known as *At*CARD1), *At*DORN1, and *At*LecRK-1.8, respectively (Chen et al., 2017a; Wang et al., 2017; Wu et al., 2020a). In tomato, the hormone peptide systemin is perceived by *Sl*SYR1/2 to enhance resistance against herbivores (Wang et al., 2018b; Figure 2D).

### PRRs involved in the recognition of parasitic plants

In addition to eH_2_O_2_, *At*CARD1 has also been shown to perceive the self-derived quinone compound 2,6-dimethoxy-1,4-benzoquinone (DMBQ; Laohavisit et al., 2020). Perception of DMBQ induces *At*CARD1-dependent immune responses. On the other hand, the parasitic plant *Phtheirospermum japonicum* perceives DMBQ via *At*CARD1 homologs *Pj*CADL1/2/3, which leads to development of haustoria for parasitic infection (Laohavisit et al., 2020). Thus, CARD1 is involved in both immunity (for nonparasitic plants) and parasitic plant infection. Plants also perceive PAMPs from parasitic plants to restrict infection. The tomato LRR–RLP *Sl*CuRe1 perceives the peptide Crip21 from the parasitic plant *Cuscuta* spp. (Hegenauer et al., 2020). Crip21 is derived from a *Cuscuta* glycine-rich cell wall protein. Activation of *Sl*CuRe1 by Crip21 elicits cell death and defense responses in tomato (Hegenauer et al., 2020; Figure 2E).

### PRRs involved in viral recognition

While some PRRs, such as *At*NIK1, have been shown to be required for viral resistance, no PRR has been reported to directly perceive viral particles (Zorzatto et al., 2015). However, the Arabidopsis PRR co-receptor *bak1* loss-of-function mutant exhibits enhanced susceptibility to multiple viruses (Kørner et al., 2013). In addition, exogenous application of double-stranded RNAs and viral coat protein (CP) elicits PTI responses in plants (Allan et al., 2001; Niehl et al., 2016). Conceivably, some uncharacterized PRR(s) are involved in the recognition of viral PAMPs (Figure 2F).

### PRRs involved in the recognition of animals

In addition to eNAD^+^, *At*LecRK-1.8 and AtLecRK-1.1 are involved in the perception of *Pieris brassicae* (cabbage moth) eggs (Gouhier-Darimont et al., 2019; Groux et al., 2021). The ligand from *P.* *brassicae* eggs that activates *At*LecRK-1.8 remains to be identified and characterized. The Arabidopsis LRR–RLK *At*NILR1 is involved in the perception of *Heterodera schachtii* (sugarbeet nematode) extracts, and *nilr1* mutants are hypersusceptible to nematode infection (Mendy et al., 2017). The cowpea (*Vigna unguiculata*) LRR–RLP *Vu*INR was shown to perceive inceptin, a proteolytic fragment of chloroplastic ATP synthase from the oral secretions of *Lepidopteran* herbivores (a HAMP; Steinbrenner et al., 2019). Whether PRRs can perceive ligands directly from herbivores remains to be determined (Figure 2G).

### PRR co-receptors

Most, if not all, PRRs function with co-receptors to activate downstream immune responses. Multiple LRR–RLKs, such as FLS2, EFR, and PEPRs function with the co-receptors *At*BAK1 and *At*BKK1 (Chinchilla et al., 2007; Roux et al., 2011). LRR–RLPs function with the co-receptors SOBIR1 and BAK1, and the LysM-RLK LYKs and LysM-RLP LYMs function with the co-receptor CERK1 (Miya et al., 2007; Willmann et al., 2011; Liebrand et al., 2013; Cao et al., 2014). These co-receptors are highly conserved in land plants and are crucial for PRR-mediated immunity (Figure 2H).

## NLRs involved in pathogen recognition

**Figure 3:**
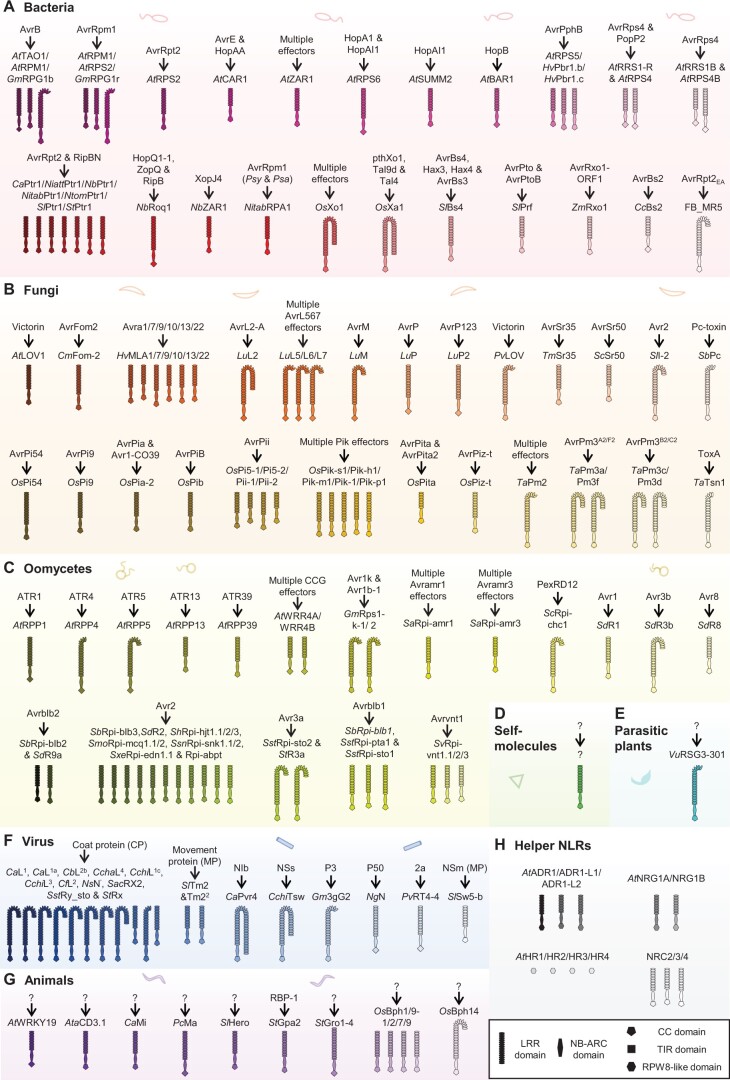
NLRs involved in plant immunity. Characterized NLRs with known effectors from (A) bacteria, (B) fungi, (C) oomycetes, (D) self-molecules, (E) parasitic plants, (F) viruses, (G) herbivores, and (H) Helper NLRs. Abbreviations for plant species: *G. max*, *Gm*; *H. vulgare*, *Hv*; *C. annuum*, *Ca*; *Nicotiana attenuate*, *Niatt*; *N. tabacum*, *Nitab*; *Nicotiana tomentosiformis*, *Ntom*; *S. tuberosum*, *St*; *Z. mays*, *Zm*; *C. chacoense*, *Cch*; *C. melo*, *Cm*; *L. usitatissimum*, *Lu*; *P. vulgaris*, *Pv*; *Triticum monococcum*, *Tm*; *S. cereale*, *Sc*; *S. bicolor*, *Sb*; *S. americanum*, *Sa*; *S. bulbocastanum*, *Sbu*; *S. chacoense*, *Sch*; *S. demissum*, *Sd*; *Solanum hjertingii*, *Sh*; *Solanum mochicense*, *Smo*; *Solanum nigrescens*, Ssn; *Solanum × edinense*, *Sxe*; *S. stoloniferum*, *Sst*; *S. venturi*, Sv; *C. baccatum*, *Cb*; *C. chinense*, *Cchi*; *C. frutescens*, *Cf*; *N. sylvestris*, *Ns*; *S. acaule*, *Sac*; *N. glutinosa*, *Ng*; *A. tauschii*, *Ata*; *P. cerasifera*, *Pc*. Number of LRR repeats in the NLRs were predicted by LRRpredictor (Martin et al., 2020a). The full list of NLRs can be found in Supplemental Data Set 2.

Sensor NLRs are involved in the recognition of effectors from viruses, bacteria, fungi, oomycetes, parasitic plants, and herbivores. Some NLRs act as helpers or co-receptors to transduce immune signals from sensor NLRs following effector recognition (Wu et al., 2018). Currently, there are more than 140 characterized NLRs with known recognized effectors (Kourelis and Kamoun, 2020). Here, we summarize a list of NLRs involved in effector recognition (Figure 3; Supplemental Data Set 2).

### NLRs involved in bacterial recognition usually act by guarding host components

Bacterial effectors have been selected that target PRR signaling components and suppress host immunity. Plants have evolved multiple NLRs to guard host immune components, which indirectly detect bacteria and induce ETI. For example, the *Pseudomonas syringae* effector AvrPto suppresses PTI by inhibiting host kinase activity (Li et al., 2005; He et al., 2006; Xing et al., 2007; Xiang et al., 2008; Wu et al., 2017b). The tomato decoy kinase Pto is guarded by the CNL Prf, which detects the perturbation of Pto kinase activity by AvrPto and activates ETI (Wu et al., 2004; Mucyn et al., 2006; Ntoukakis et al., 2013). Since plants have evolved multiple NLRs to guard central immune signaling pathways, some effectors from *P.* *syringae* are recognized by multiple NLRs from different plant species (Jones and Dangl, 2006). Examples include the following: AvrB is recognized by *At*TAO1, *At*RPM1, and *Glycine max Gm*RPG1b (Grant et al., 1995; Ashfield et al., 2004; Eitas et al., 2008). AvrRpm1 from *P*. *syringae* pv. *maculicola* (*Pma*) is recognized by *At*RPM1, *At*RPS2, and *Gm*RPG1r (Ashfield et al., 1995; Grant et al., 1995; Kim et al., 2009a). AvrPphB is recognized by *At*RPS5, *Hordeum vulgare Hv*Pbr1.b and *Hv*Pbr1.c (DeYoung et al., 2012; Carter et al., 2019; Laflamme et al., 2020). AvrRpt2 from *P.* *syringae* and RipBN from *Ralstonia pseudosolanacearum* are recognized by the CNL Ptr1 from multiple Solanaceous species (Mazo-Molina et al., 2020). In addition, AvrRpt2 is recognized by the CNL *At*RPS2, and AvrRpt2_EA from *Erwinia amylovora* is recognized by FB_MR5 from *Malus × robusta 5* (Axtell and Staskawicz, 2003; Mackey et al., 2003; Peil et al., 2019). HopA1 is recognized by *At*RPS6, and HopAI1 is recognized by both *At*SUMM2 and *At*RPS6 (Kim et al., 2009b; Zhang et al., 2012; Takagi et al., 2019).

On the other hand, central hubs of the immune system are targeted by multiple effectors. Correspondingly, NLRs, which guard central immune signaling components, can recognize multiple effectors (Khan et al., 2016). For example, the CNL *At*ZAR1 functions with the pseudokinase RKS1 to guard the receptor-like cytoplasmic kinase (RLCK) PBL2 (Wang et al., 2015). By guarding RLCKs or decoy pseudokinases, *At*ZAR1 indirectly recognizes HopZ1a, HopF2, HopBA1, HopO1, HopX1, and AvrAC from *P*. *syringae* or *Xanthomonas campestris*, and potentially more effectors that target RLCKs (Wang et al., 2015; Laflamme et al., 2020). *Nb*ZAR1 is also required to recognize XopJ4 from *Xanthomonas perforans* via the pseudokinase JIM2 (Schultink et al., 2019). Other examples include the following: AvrRpm1_Psa_ and AvrRpm1_Psy_ from *P*. *syringae* pv.* actinidiae biovar 3* (*Psa*) and *P*. *syringae* pv. *syringae strain B728a* (*Psy*) are recognized by *Nicotiana tabacum Nitab*RPA1 (Yoon and Rikkerink, 2020). AvrE and HopAA are both recognized by Arabidopsis CAR1 (Laflamme et al., 2020). AvrRps4 from *P*. *syringae* pv. *pisi* and PopP2 from *Ralstonia solanacearum* are recognized by the paired-TNLs *At*RRS1-R and *At*RPS4 (Narusaka et al., 2009; Sarris et al., 2015). In addition, AvrRps4 can also be recognized by the paired-TNLs *At*RRS1B and *At*RPS4B (Saucet et al., 2015). The TNL *Nb*Roq1 recognizes HopQ1-1, XopQ, and RipB from *P*. *syringae*, *Xanthomonas*, and *R.* *solanacearum*, respectively (Schultink et al., 2017; Thomas et al., 2020). Multiple TRANSCRIPTION ACTIVATOR-LIKE (TAL) effectors from *Xanthomonas oryzae* are recognized by the CNLs *Os*Xo1 and *Os*Xa1 (Yoshimura et al., 1998; Triplett et al., 2016; Read et al., 2020a, 2020b).The tomato TNL *Sl*Bs4 also recognizes multiple *Xanthomonas* effectors (Schornack et al., 2004, 2005). AvrRxo1-ORF1 from *X.* *oryzae* and *Burkholderia andropogonis* are recognized by the CNL *Zm*Rxo1 from maize (*Zea mays*; Zhao et al., 2004; Figure 3A).

### NLRs involved in fungal recognition

Plant NLRs recognize multiple effectors and molecules from fungal pathogens. Victorin, a secondary metabolite from *Cochliobolus victoriae*, is recognized by LOV1 from Arabidopsis and *Phaseolus vulgaris* (Sweat et al., 2008; Lorang et al., 2018). AvrFom2 from *Fusarium oxysporum* is recognized by the CNL *Cm*Fom-2 from *Cucumis melo* (Schmidt et al., 2016). *Hordeum vulgare* RESISTANCE LOCUS A NLRs recognize a range of effectors from *Blumeria graminis* (Ridout et al., 2006; Lu et al., 2016; Saur et al., 2019) and can even recognize races of wheat stripe rust (Bettgenhaeuser et al., 2021). Multiple TNLs from *Linum usitatissimum* recognize effectors from *Melampsora lini* (Dodds et al., 2004; Dodds and Thrall, 2009; Catanzariti et al., 2010; Anderson et al., 2016). Effectors from the rice blast fungus *Magnaporthe oryzae* are recognized by multiple CNLs from *O.* *sativa* (Jia et al., 2000; Ashikawa et al., 2008, 2012; Li et al., 2009, 2019; Zeng et al., 2011; Rai et al., 2011; Sone et al., 2013; Zhai et al., 2014; Devanna et al., 2014; Zhang et al., 2015; Wu et al., 2015; Vo et al., 2019). Effectors from *B.* *graminis*, *P.* *nodorum*, *Pyrenophora tritici-repentis*, and *Puccinia graminis* are recognized by multiple CNLs from *Triticum* species (Srichumpa et al., 2005; Liu et al., 2006; Salcedo et al., 2017; Bourras et al., 2019; Navathe et al., 2020; Manser et al., 2021). AvrSr50 from *P.* *graminis* is recognized by *Sc*Sr50 from *Secale cereale* (Chen et al., 2017b). Avr2 from *F.* *oxysporum* is recognized by the CNL *Sl*I2, and Pc-toxin from *Periconia circinata* is recognized by the CNL *Sb*Pc from *Sorghum bicolor* (Nagy et al., 2007; Nagy and Bennetzen, 2008; Houterman et al., 2009; Figure 3B).

### NLRs involved in the recognition of oomycetes

Multiple effectors from *Hyaloperonospora arabidopsidis* (*Hpa*) are recognized by Arabidopsis NLRs. ATR1, ATR4, ATR5, ATR13, and ATR39 are recognized by *At*RPP1, *At*RPP4, *At*RPP5, *At*RPP13, and *At*RPP39, respectively (Rentel et al., 2008; Krasileva et al., 2010; Bailey et al., 2011; Goritschnig et al., 2012; Asai et al., 2018). CX2CX5G effector-like proteins (CCG effectors) from *Albugo candida* are recognized by *At*WRR4A and *At*WRR4B (Redkar et al., 2021).

The oomycete genus *Phytophthora* carries multiple phytopathogenic species that cause enormous crop losses worldwide. Identification of NLRs that recognize *Phytophthora* effectors provides resources for crop resistance. The *P.* *sojae* effectors Avr1k and Avr1b-1 are recognized by *Gm*Rps1-k (Song et al., 2013). Effectors from *P. infestans* are also recognized by NLRs from multiple *Solanaceae* species. For example, the effectors Avramr1 and Avramr3, with homologs in many *Phytophthora* species, are recognized by Rpi-amr1 (from *Solanum* *americanum*) and Rpi-amr3, respectively (Lin et al., 2020, 2021; Witek et al., 2021). Avrblb1 is recognized by Rpi-blb1 (from *Solanum* *bulbocastanum*), Rpi-pta1, and Rpi-sto1 (from *Solanum* *stoloniferum*; Vleeshouwers et al., 2008; Oh et al., 2009). Avrblb2 is recognized by Rpi-blb2 and R9a (from *S. bulbocastanum* and *Solanum* *demissum*, respectively; Oh et al., 2009; Jo, 2013). PexRD12 is recognized by Rpi-chc1 (from *Solanum* *chacoense*; Monino-Lopez et al., 2021; Petre et al., 2021). Avr1, Avr3b, and Avr8 are recognized by R1, R3b, and R8, respectively (Ballvora et al., 2002; Li et al., 2011; Jo, 2013; Du et al., 2015a; Vossen et al., 2016). *Pi*Avr2 is recognized by multiple NLRs from *Solanaceae* (Park et al., 2005; Lokossou et al., 2009; Champouret, 2010; Aguilera-Galvez et al., 2018). Avr3a is recognized by Rpi-sto2 and R3a (from *Solanum* *tuberosum*; Bos et al., 2010; Champouret, 2010; Vleeshouwers et al., 2011; Chapman et al., 2014). Avrvnt1 is recognized by Rpi-vnt1 from *Solanum* *venturi* (Foster et al., 2009; Pel, 2010; Figure 3C).

### Apparent absence of NLRs involved in self-recognition in plants

In mammals, DAMPs can be indirectly recognized the intracellular NOD-, LRR-, and pyrin domain-containing protein 3-inflammasome in macrophages (Swanson et al., 2019). However, no plant NLRs have been reported to detect self-molecules so far (Figure 3D).

### NLRs involved in the recognition of parasitic plants

Virus-induced silencing of the CNL *Vu*RSG3-301 from *V.* *unguiculata* leads to enhanced susceptibility to the parasitic plant *Striga gesnerioides* race 3 (Li and Timko, 2009). The effector recognized by *Vu*RSG3-301 has not yet been identified (Figure 3E).

### NLRs involved in viral recognition

The CPs from different viruses are recognized by pepper (*Capsicum annuum*) *Ca*L^1^, *Ca*L^1a^, *Capsicum baccatum Cb*L^2b^, *Capsicum chacoense Ccha*L^4^, *Capsicum chinense Cchi*L^1c^, *Cchi*L^3^, *Capsicum frutescens Cf*L^2^, *Nicotiana sylvestris Ns*N^′^, *Solanum acaule* Rx2, *S.* *stoloniferum* Ry_sto_, and potato (*S.* *tuberosum*) Rx (Saito et al., 1987; Bendahmane et al., 1995; Berzal-Herranz et al., 1995; Gilardi et al., 2004; Tameling and Baulcombe, 2007; Matsumoto et al., 2008; Tomita et al., 2011; Mizumoto et al., 2012; Grech-Baran et al., 2021). Viral movement proteins are recognized by Tm2, *Sl*Tm2^2^, and *Sl*Sw5-b (Pelham, 1966; Hall, 1980; Weber and Pfitzner, 1998; Peiró et al., 2014). The RNA-dependent RNA Polymerase (NIb) of potyviruses is recognized by the *Ca* Pvr4 (Kim et al., 2015). The RNA silencing suppressor protein NSs from tomato spotted wilt virus is recognized by *Cchi*Tsw (de Ronde et al., 2013). P3 cistrons from soybean mosaic virus are recognized by *Gm*3gG2 (Wen et al., 2013). The helicase domain of the tobacco mosaic virus replicase (p50) is recognized by *Nicotiana glutinosa N* (Whitham et al., 1994; Erickson et al., 1999). Cucumber mosaic virus 2a protein is recognized by *P.* *vulgaris Pv*RT4-4 (Seo et al., 2006). To summarize, multiple components involved in the process of viral infection are recognized by NLRs (Figure 3F).

### NLRs involved in the recognition of animals

Multiple NLRs were shown to be involved in resistance against herbivores. NLRs involved in nematode resistance include the TIR–NB–LRR pair *At*DSC1 and *At*WRKY19, *Aegilops tauschii Ata*CD3.1, *Ca*Mi, *Prunus cerasifera Pc*Ma, *Sl*Hero, *St*Gpa-2, and *St*Gro1-4 (Lagudah et al., 1997; van der Voort et al., 1997; Milligan et al., 1998; Paal et al., 2004; Sobczak et al., 2005; Chen et al., 2007; Claverie et al., 2011; Warmerdam et al., 2020). In addition, the tomato *Mi* gene confers resistance to multiple herbivores, such as nematodes, aphids, and whiteflies (Kaloshian et al., 1995; Milligan et al., 1998; Rossi et al., 1998; Neiva et al., 2019). Other NLRs have been shown to confer resistance against the arthropod *Nilaparvata lugens* (brown planthopper). These include the rice *Os*Bph1/9 and *Os*Bph14 (Du et al., 2009; Zhao et al., 2016). While multiple NLRs are involved in herbivore resistance, more work is needed to identify the recognized effectors (Figure 3G).

### Helper NLRs

While some sensor NLRs do not require helper NLRs, many NLRs function with helper NLRs to transduce immune signals. In Arabidopsis, some CNLs and/or most TNLs require the RNLs ACTIVATED DISEASE RESISTANCE 1 (collectively known as ADR1s, which includes *At*ADR1, *At*ADR1-L1, and *At*ADR1-L2) and/or N REQUIREMENT GENE 1 (collectively known as NRG1s, which includes *At*NRG1A and *At*NRG1B; Bonardi et al., 2011; Castel et al., 2019a; Wu et al., 2019; Saile et al., 2020). In Arabidopsis accession Col-0, the four RPW8 homologs, *At*HR1, *At*HR2, *At*HR3, and *At*HR4, also contribute to resistance against bacterial and fungal pathogens (Barragan et al., 2019; Castel et al., 2019b). In Solanaceous plants, the CNLs NB-LRR REQUIRED FOR HR-ASSOCIATED CELL DEATH-2 (NRC2), NRC3, and NRC4 function as helper NLRs for multiple sensor NLRs (Wu et al., 2017a; Figure 3H). The contribution of the NRC network to the functions of sensor NLRs has been extensively discussed (Wu et al., 2018; Ngou et al., 2021c).

## The PRR signaling pathway

The extracellular domains of plant PRRs perceive diverse ligands (Boutrot and Zipfel, 2017). Binding of ligands leads to heterodimeric receptor complex formation between PRRs and their co-receptors, such as BAK1 and CERK1 (Miya et al., 2007; Ma et al., 2016; Hohmann et al., 2017). On the other hand, RLPs constitutively interact with SOBIR1 and recruit BAK1 upon ligand recognition (Liebrand et al., 2013; Albert et al., 2015). In Arabidopsis, the bacterial flagellin peptide flg22 is perceived by the LRR–RLK FLS2 (Felix et al., 1999; Chinchilla et al., 2006). Flg22 acts as a “molecular glue” and interacts with and brings together the extracellular LRR domains of FLS2 and BAK1 (Sun et al., 2013; Hohmann et al., 2017). Heterodimeric complex formation between the LRR domains of FLS2 and BAK1 brings their cytoplasmic kinase domains into close proximity, which leads to a series of auto- and trans-phosphorylation events (Schwessinger et al., 2011; Cao et al., 2013; Sun et al., 2013). This activated receptor complex then phosphorylates RLCKs (Lin et al., 2013; Liang and Zhou, 2018). RLCK subfamily VII members (collectively known as RLCK-VIIs) were first shown to be important for surface receptor-mediated immunity in tomato and tobacco and to be required for *Cf-4* and *Cf-9* to confer fungal resistance (Rowland et al., 2005). In Arabidopsis, RLCKs play particularly important roles during PRR-mediated immunity (Lu et al., 2010; Lin et al., 2014; Liang and Zhou, 2018; Rao et al., 2018). BAK1 associates with and phosphorylates the RLCK-VII BIK1 at the Try243 and Try250 residues (Lu et al., 2010; Lin et al., 2014).

**Figure 4:**
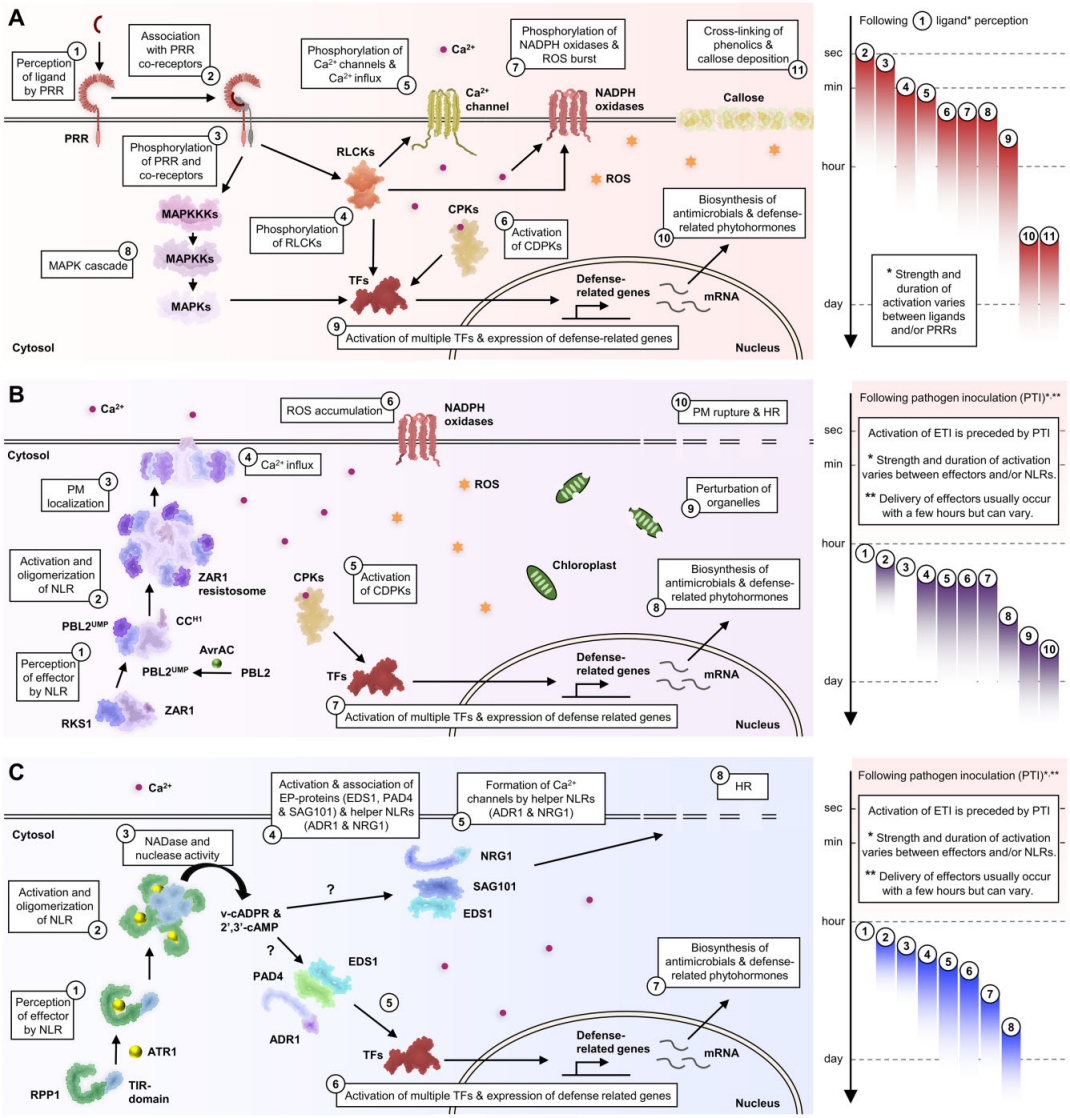
Plant immune signaling pathways. A, PRR signaling pathway. Ligand perception by PRRs activates multiple kinases, which leads to calcium influx to the cytosol, ROS production, transcriptional reprogramming, and callose deposition. B, Singleton NLR signaling pathway. The ZAR1/RKS1 heterodimer detects the effector AvrAC via association with uridylylated PBL2 by AvrAC. This leads to the activation and oligomerization of ZAR1. The ZAR1 resistosome localizes to the PM and triggers calcium influx, which leads to the HR and cell rupture. C, Helper-NLR-dependent sensor NLR signaling pathway. Recognition of ATR1 by the TNL RPP1 leads to oligomerization and the induced proximity of TIR domains. The TIR domain exhibits NADase activity and produces v-cADPR, which might activate EP-proteins and the helper NLRs (RNLs). Following TNL activation, EP-proteins and RNLs associate with each other and activate downstream immune responses, likely via cation channel activity from the helper NLRs. Timeline on the right indicates the order and duration of each signaling event following ligand/effector perception. Numbers indicate the corresponding signaling events in the figure on the left. Note that the activation of ETI is usually preceded by PTI activation, and the strength and duration of each event vary and are dependent on the PRRs/NLRs that are activated.

The activation of RLCK-VIIs promotes the phosphorylation of multiple signaling components, including the calcium channels CNGC2/4 and OSCA1.3, the NADPH oxidase respiratory burst oxidase protein D (RbohD), and the mitogen-activated protein kinase kinase kinase (MAPKKK5) (Kadota et al., 2014; Li et al., 2014; Bi et al., 2018; Tian et al., 2019; Thor et al., 2020). The activation of multiple calcium channels by BIK1 leads to cytosolic calcium influx, which activates calcium-dependent protein kinases (CPKs). In Arabidopsis, CPK4/5/6/11, together with BIK1, phosphorylate and activate RbohD, which leads to reactive oxygen species (ROS) production (Kadota et al., 2014, 2015; Li et al., 2014). The phosphorylation of multiple ion channels by RLCKs also leads to stomatal closure in response to PAMPs (Liu et al., 2019; Thor et al., 2020). In parallel, MAPKKK3 and MAPKKK5 phosphorylate the MAPKKs MKK4 and MKK5, which then phosphorylate the MAPKs MPK3 and MPK6 in Arabidopsis. In parallel, MKK1/MKK2 also phosphorylate MPK4 (Asai et al., 2002; Rasmussen et al., 2012). RLCK-VIIs, CPKs, and MPKs phosphorylate and activate multiple defense-related transcription factors, such as WRKY transcription factors, resulting in the upregulation of defense-related genes (Boudsocq et al., 2010; Gao et al., 2013; Lal et al., 2018). PTI-induced transcriptional reprogramming leads to the biosynthesis of antimicrobial compounds and defense-related hormones, such as ET and salicylic acid (SA; Macho et al., 2014; Bigeard et al., 2015; Guan et al., 2015; Bjornson et al., 2021). Hydrogen peroxide (a type of ROS) promotes protein and phenolic cross-linking, which result in callose deposition and restricts fungal and oomycete infection (Luna et al., 2011; Voigt, 2014; Figure 4A).

## Signaling pathway of singleton NLRs

NLR-mediated immunity is triggered by the detection of effectors through intracellular NLRs. NLRs detect effectors either via direct interactions with effectors, guarding effector targets, or guarding decoy proteins (Van der Biezen and Jones, 1998; Dangl and Jones, 2001; van der Hoorn and Kamoun, 2008). In Arabidopsis, CNLs and TNLs act as sensor NLRs that recognize effectors, while RNLs act as helper NLRs to transduce immune signals (Feehan et al., 2020). While the majority of sensor NLRs in Arabidopsis require helper NLRs to mediate immunity, some CNLs mediate immune responses alone. These are known as singleton NLRs, such as ZAR1 and RPM1 (Adachi et al., 2019b). ZAR1 recognizes a range of effectors by monitoring pseudokinases such as RKS1 and PBL2, which mimic authentic RLCK targets of effectors (Wang et al., 2019a). The bacterial effector AvrAC from *X.* *campestris* uridylylates the RLCK PBL2. The ZAR1/RKS1 heterodimer associates with uridylylated PBL2 (PBL2^UMP^), which leads to conformational changes in the heterodimer. ADP in the NB-ARC domain in ZAR1 is ejected and replaced by ATP (Wang et al., 2019b). This results in the oligomerization of ZAR1/RKS1/PBL2^UMP^ oligomers into pentameric resistosomes (Wang et al., 2019a) that localize to the PM to trigger downstream immune responses (Wang et al., 2019a; Bi et al., 2021).

ZAR1 resistosomes were recently shown to exhibit cation channel activity (Bi et al., 2021). The N-terminal α-helices in ZAR1 form a funnel-shaped structure with a negatively charged carboxylate ring, which allows cations to pass through into the cytosol. Co-expression of ZAR1 with RKS1, PBL2, and AvrAC in plant protoplasts results in cytosolic calcium influx, ROS accumulation, and the perturbation of chloroplasts and vacuoles (Bi et al., 2021). Robust ROS accumulation during ZAR1 activation is likely caused by the activation of multiple downstream signaling components, such as the NADPH oxidases, since the CPKs are activated by cytosolic calcium influx (Gao et al., 2013). In addition, multiple CPKs and RbohD have been shown to be phosphorylated during RPS2 activation (Gao et al., 2013; Kadota et al., 2019). Defense-related transcription factors are also likely activated by cytosolic calcium influx (Boudsocq et al., 2010; Gao et al., 2013). The perturbation of chloroplasts and vacuoles is quickly followed by the loss of PM integrity and cellular rupture (Bi et al., 2021; Figure 4B). How these processes are regulated by immune signaling components and their relationships to transcriptional reprogramming are currently unclear.

## The signaling pathway of helper-NLR-dependent sensor NLRs

The majority of sensor NLRs requires helper NLRs to mediate immunity. In solanaceous plants, the NB-LRR REQUIRED FOR HR-ASSOCIATED CELL DEATH proteins (collectively known as NRCs) are required for immunity and hypersensitive cell death response (HR) mediated by multiple sensor NLRs (Wu et al., 2017a). Interestingly, the N-terminal CC domain in ZAR1 contains a “MADA motif” that is also present in NRCs (Adachi et al., 2019a). This suggests that perhaps NRCs also form cation channels with α-helices following activation. In Arabidopsis, ADR1s and NRG1s are required for resistance and HR mediated by some CNLs and many TNLs (Bonardi et al., 2011; Castel et al., 2019a; Wu et al., 2019; Saile et al., 2020). Following effector recognition, TNLs also oligomerize into resistosomes to mediate resistance (Ma et al., 2020a; Martin et al., 2020). The Arabidopsis RPP1 recognizes the *Hpa* effector ATR1, and *N. benthamiana* ROQ1 recognizes the *Xanthomonas* effector XopQ. These effectors are recognized by the LRR and post-LRR domain, which likely leads to conformational changes and oligomerization of these TNLs into tetrameric resistosomes (Ma et al., 2020a; Martin et al., 2020).

The TIR domains of TNLs are brought into close proximity following oligomerization, activating NADase activity and producing v-cADPR (Horsefield et al., 2019; Wan et al., 2019a; Duxbury et al., 2020; Ma et al., 2020a; Martin et al., 2020). TIR domains also exhibit 2′,3′-cAMP/cGMP synthetase activity by hydrolyzing RNA or DNA (Yu et al., 2021). v-cADPR and 2′,3′-cAMP/cGMP are proposed to be signaling molecules that activate downstream signaling components (Horsefield et al., 2019; Wan et al., 2019a; Yu et al., 2021). Following the activation of TNLs, the EP-domain containing proteins (EP-proteins) SAG101 and EDS1 associate with NRG1 (Sun et al., 2021). Similarly, the activation of TNLs also leads to the association of the EP-proteins PAD4 and EDS1 with ADR1 (Wu et al., 2021b). These associations lead to the activation of these signaling components, which in turn activate downstream immune responses, such as defense-related gene expression and HR (Lapin et al., 2019; Sun et al., 2021). The RNLs ADR1 and NRG1 were also recently shown to function as calcium channels to activate immunity (Jacob et al., 2021). It is conceivable that the association and activation of helper RNLs and EP-proteins induces calcium influx and triggers downstream immune responses (Figure 4C).

## Physiological responses induced by RLKs

**Figure 5:**
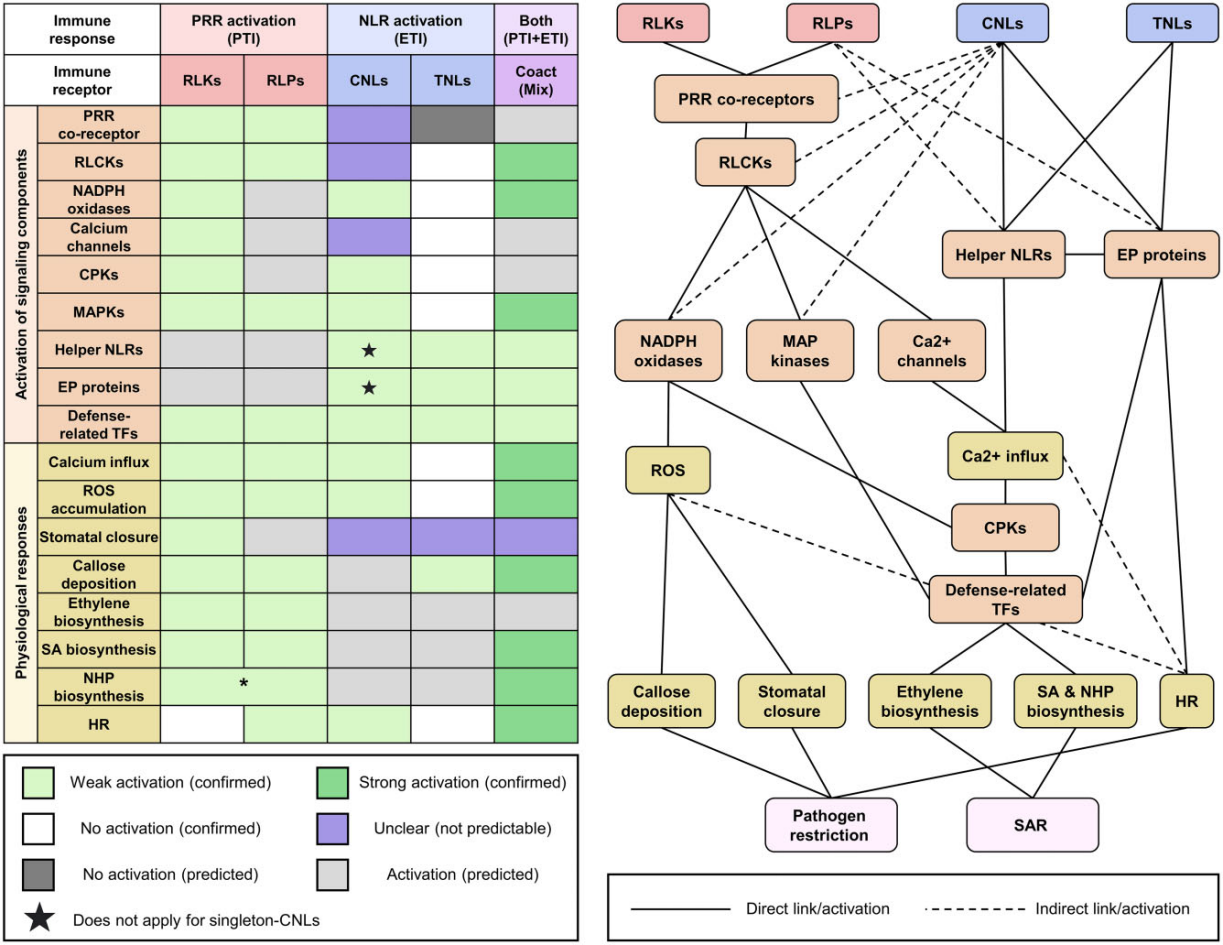
Signaling components and physiological responses activated by different modes of action of immune receptors. (Left) Tabular summary of signaling components and physiological responses activated by RLKs, RLPs, CNLs, TNLs, and coactivation of PRRs and NLRs. Green (weak or strong activation) and white (no activation) shading represent confirmed responses from publications. Gray shading indicates predicted responses. Purple shading represents unclear responses that cannot be predicted. Asterisks indicate inoculation with the bacterial pathogen *P. syringae* pv. *maculicola* (*Psm*) leads to NHP accumulation (Wang et al., 2018c; Liu et al., 2020). (Right) PRR and NLR signaling network. Activation of PRRs (red) and NLRs (blue) lead to the activation of downstream signaling components (orange) and physiological responses (yellow), which result in resistance against pathogens (pink). Note that the activation of physiological responses can vary between immune receptors and are dependent on specific PRRs/NLRs.

Following ligand perception, the PRR co-receptor BAK1 and the RLCK BIK1 are phosphorylated (Lin et al., 2014; Perraki et al., 2018). This leads to the phosphorylation and activation of multiple signaling components (Macho and Zipfel, 2014). The activation of multiple calcium channels and NADPH oxidases leads to calcium influx, stomatal closure, ROS production, and callose deposition (Luna et al., 2011; Kadota et al., 2014; Li et al., 2014; Thor et al., 2020). The activation of CPKs and MAPKs leads to transcriptional reprograming and the biosynthesis of defense-related hormones (Boudsocq et al., 2010). In Arabidopsis, MPK3/MPK6 activate 1-AMINOCYCLOPROPANE-1-CARBOXYLIC ACID SYNTHASE (ACS) isoforms ACS2 and ACS6, which are involved in ET biosynthesis (Liu and Zhang, 2004; Han et al., 2010). The transcription factors SYSTEMIC-ACQUIRED RESISTANCE DEFICIENT 1 (SARD1) and CALMODULIN-BINDING PROTEIN 60 G (CBP60g) are required for PTI-induced upregulation of SA biosynthesis genes, such as *ISOCHORISMATE SYNTHASE 1* (*ICS1*), *EDS5*, and *AVRPPHB SUSCEPTIBLE 3* (*PBS3*; Zhang et al., 2010b; Sun et al., 2015). SARD1 and CBP60g are also required for the upregulation of pipecolic acid (*N*-hydroxyl-pipecolic acid [NHP])-biosynthesis genes, such as *FLAVIN-CONTAINING MONOOXYGENASE 1* (Sun et al., 2015; Liu et al., 2020; Figure 5).

## Physiological responses induced by RLPs

Similar to RLKs, RLPs also require PRR co-receptors, RLCKs, CPKs, and MAPKs to transduce immune signals (Piedras et al., 1998; Romeis et al., 1999, 2000; Rowland et al., 2005; González-LamotHe et al., 2006; Yang et al., 2006; van den Burg et al., 2008). In Arabidopsis, nlp20-induced immune responses mediated by RLP23 require the co-receptors BAK1, SOBIR1, and multiple RLCKs such as PBL19/20/30/31/32 (Albert et al., 2015; Pruitt et al., 2021; Tian et al., 2020). The activation of RLP23 leads to changes in PM potential, an ROS burst, the phosphorylation of BIK1 and MAPKs, callose deposition, and SA and ET production, similar to the activation of FLS2 (Wan et al., 2019b). In addition, flg22 and nlp20 induce highly overlapping transcriptional reprogramming in Arabidopsis (Wan et al., 2019b; Bjornson et al., 2021). Thus, RLKs and RLPs induce overlapping responses due to the activation of similar downstream signaling components. However, the individual activation of multiple RLPs, such as *Sl*Cf-4, *Sl*Cf-9, and *At*RLP23, leads to the HR, perhaps due to the prolonged activation of downstream signaling components (Jones et al., 1994; Thomas et al., 1997; Rowland et al., 2005; Albert et al., 2015). PAD4, EDS1, and ADR1 are required for both RLK- and RLP-mediated immunity (Pruitt et al., 2021; Tian et al., 2021). Thus, EP-proteins and helper NLRs might also be activated during some PTI signaling, although it remains to be established whether EP proteins play a primary or secondary role in defense signaling (Figure 5).

## Physiological responses induced by CNLs alone

Activation of the Arabidopsis CNL RPS2 in the absence of PTI leads to the phosphorylation of RbohD (in Ser343/347), CPKs, and MAPKs (Gao et al., 2013; Tsuda et al., 2013; Kadota et al., 2019; Ngou et al., 2021a; Yuan et al., 2021). RPS2-induced RbohD phosphorylation and ROS production are dependent on BAK1/BKK1 and BIK1 (Yuan et al., 2021). However, it is currently unclear whether BAK1/BKK1 and BIK1 are directly or indirectly activated by CNLs. While the ZAR1 resistosome directly triggers calcium influx, other calcium channels may also be activated by CNLs (Bi et al., 2021). The activation of RPM1, RPS2, and RPS5 leads to MAPK activation and the HR (Ngou et al., 2021a). In addition, the activation of many CNLs leads to the upregulation of SA- and NHP-biosynthesis genes (Jacob et al., 2018; Ngou et al., 2021a). Thus, ET, SA, and NHP are likely to be produced during CNL activation (Figure 5).

## Physiological responses induced by TNLs alone

Activation of the Arabidopsis TNL RRS1/RPS4 does not lead to the phosphorylation of BIK1, RbohD (in Ser39/343/347), MAPKs, calcium influx, ROS accumulation, or the HR (Ngou et al., 2020, 2021a). Thus, RLCKs, NADPH oxidases, calcium channels, or CPKs are unlikely to be activated by RRS1/RPS4 alone. Activation of RRS1/RPS4 induces weak callose deposition, perhaps via SA accumulation (Tateda et al., 2014; Ngou et al., 2021a). Activation of TNLs leads to the association of EP-proteins with helper NLRs, which induces transcriptional reprogramming (Saile et al., 2020; Sun et al., 2021; Wu et al., 2021b). Similar to CNLs, the activation of TNLs leads to the upregulation of SA- and NHP-biosynthesis genes (Ding et al., 2020; Ngou et al., 2021a). Thus, SA and NHP are likely to be produced during TNL activation (Figure 5).

## Physiological responses induced by the co-activation of PRRs and NLRs

Co-activation of PRRs and NLRs (“PTI + ETI”) leads to the robust activation of BIK1, RbohD, and MPK3 (Tsuda et al., 2013; Su et al., 2018; Ngou et al., 2021a; Yuan et al., 2021). This results in stronger calcium influx, ROS accumulation, and callose deposition compared to PTI or ETI alone (Ngou et al., 2021a; Yuan et al., 2021). In addition, “PTI + ETI” leads to stronger accumulation of SA and NHP compared to PTI alone, which is likely due to the stronger expression of SA- and NHP-biosynthesis genes during ETI (Wang et al., 2018c; Castel et al., 2019a; Ding et al., 2020; Liu et al., 2020; Figure 5).

## Regulation of PRR-mediated immunity

The PRR-signaling pathway is tightly regulated as the excessive activation of PRRs leads to autoimmunity and growth inhibition (Navarro et al., 2006; Albrecht et al., 2012; Huot et al., 2014).

### Regulation of PRRs

**Figure 6:**
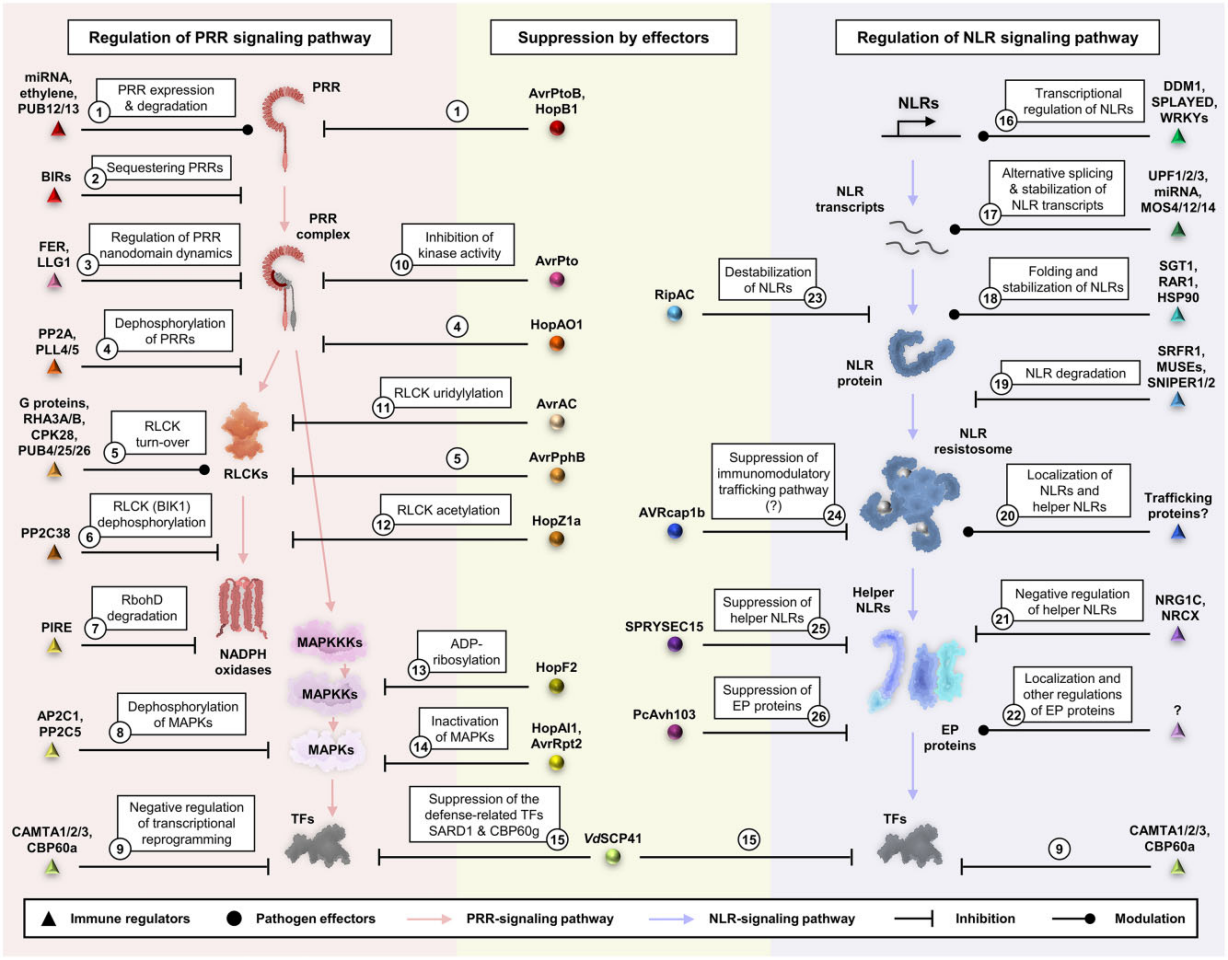
Regulation and suppression of immunity by plant proteins and pathogen-derived effectors. (Left; red shading) regulation of the PRR signaling pathway by host proteins. Protein abundance and PTMs of PRRs and PRR signaling components are tightly regulated. (Middle; yellow shading) suppression of immunity by pathogen effectors. Many identified effectors suppress PTI via multiple mechanisms. Very few effectors that target the NLR signaling pathway have been identified so far. (Right; blue shading) regulation of the NLR signaling pathway by host proteins. Both the transcript and protein level of NLRs are tightly regulated by multiple processes. The regulation of signaling events post-NLR activation has not been well characterized. Numbers indicate the corresponding mechanisms of immune regulation.

Both the transcript and protein levels of PRRs are regulated by multiple mechanisms. For example, the expression of *FLS2* is regulated by the microRNA miR172b (Zou et al., 2018). The expression of *FLS2* is also upregulated by ET (Boutrot et al., 2010). U-BOX DOMAIN-CONTAINING PROTEIN 12 (PUB12) and PUB13 mediate the polyubiquitination of FLS2, which leads to the endocytosis and degradation of this protein (Lu et al., 2011). Cf-4 also undergoes endocytosis upon Avr4 recognition (PostMa et al., 2016). The activation of PRRs and their co-receptors must also be regulated. BAK1-INTERACTING RECEPTOR (BIR)-LIKE KINASE 1 is an RLK that associates with and sequesters BAK1 to prevent the auto-activation of BAK1-associated PRRs (Gao et al., 2009; Ma et al., 2017; Hohmann et al., 2018). Following PAMP perception, the peptide RAPID ALKALINIZATION FACTOR 23 (RALF23) is perceived by a PRR complex composed of the *Cr*RLK1L FERONIA (FER) and the LORELEI-LIKE-GPI ANCHORED PROTEIN 1. The perception of RALF23 by FER negatively regulates the formation of the FLS2–BAK1 complex (Stegmann et al., 2017; Xiao et al., 2019). FER regulates PM nanodomain organization to modulate PRR signaling (Gronnier et al., 2020). In addition, the phosphorylation status of PRRs is regulated by multiple protein phosphatases. In Arabidopsis, POLTERGEIST-LIKE 4 (PLL4) and PLL5 associate with EFR and negatively regulate elf18-induced responses (Holton et al., 2015). PROTEIN PHOSPHATASE 2A negatively regulates the phosphorylation status of BAK1 (Segonzac et al., 2014; Figure 6).

### Regulation of PRR-signaling components

In addition to PRRs, downstream signaling components are also regulated to prevent prolonged activation. As a central signaling component in the PRR-signaling pathway, the Arabidopsis RLCK BIK1 is regulated by multiple mechanisms. EXTRA-LARGE G PROTEIN 2 (XLG2) functions with other heterotrimeric G proteins to attenuate proteasome-mediated degradation of BIK1 (Liang et al., 2016). The turnover of BIK1 is regulated by CPK28, PUB4/25/26, and the E3 ubiquitin ligases RING-H2 FINGER A3A/B (Monaghan et al., 2014; Wang et al., 2018a; Derkacheva et al., 2020; Ma et al., 2020b). The phosphorylation status of BIK1 is also negatively regulated by the protein phosphatase PP2C38 (Couto et al., 2016). In addition to RLCKs, other PRR-signaling components must also be regulated. RbohD is ubiquitinated by the E3 ubiquitin ligase PIRE (PBL13 interacting RING domain E3 ligase), which leads to proteasome-mediated degradation (Lee et al., 2020). PHAGOCYTOSIS OXIDASE/ BEM1P (PB1) DOMAIN-CONTAINING PROTEIN negatively regulates ROS production by controlling the localization of RbohD (Goto et al., 2020). The PP2C phosphatases PP2C5 and AP2C1 negatively regulate the phosphorylation of MPK3 and MPK6 (Brock et al., 2010; Figure 6).

## Regulation of NLR-mediated immunity

Similar to PRRs, the prolonged activation of NLRs also leads to autoimmunity. Thus, the regulation of both NLRs and downstream signaling components is important to prevent autoimmunity.

### Regulation of NLRs

The expression of NLRs is regulated at multiple levels (van Wersch et al., 2020). The transcription of *NLRs* is regulated by chromatin-remodeling proteins such as DECREASE IN DNA METHYLATION 1, SWI/SNF CHROMATIN REMODELER SYD, and multiple WRKY transcription factors (Li et al., 2010b; Johnson et al., 2015; Lai and Eulgem, 2018). *NLR* transcript stability is also regulated by microRNAs and NONSENSE-MEDIATED mRNA DECAY factors, such as UP-FRAMESHIFT1/2/3 (Shivaprasad et al., 2012; Jung et al., 2020). *NLR* transcripts also undergo alternative splicing, which is regulated by some MODIFIER OF SUPPRESSOR OF NPR1-1 (SNC1; MOS) proteins such as MOS4/12/14 (Zhang and Gassmann, 2007; Xu et al., 2011, 2012).

REQUIRED FOR MLA12 RESISTANCE 1 (RAR1), SUPPRESSOR OF THE G2 ALLELE OF SKP1 (SGT1), and HEAT SHOCK PROTEIN 90 (HSP90) function together as protein chaperones to regulate the folding, localization, and turnover of NLRs (Azevedo et al., 2002; Peart et al., 2002; Takahashi et al., 2003; Shirasu, 2009). In addition, NLR protein turnover is regulated by the SGT1-interacting protein SUPPRESSORS OF RPS4-RLD, multiple MUTANT SNC1-ENHANCING proteins, and the E3 ligases SNIPER1 and SNIPER2 (Li et al., 2010a; Huang et al., 2016; Dong et al., 2018; Wu et al., 2020c).

The localization of the ZAR1 resistosome to the PM is required for ZAR1-mediated resistance (Wang et al., 2019a; Bi et al., 2021). In addition, the Arabidopsis importin-α nuclear transport receptor protein IMP-α3/MOS6 is required for SUPPRESSOR OF SNC1-mediated immunity (Lüdke et al., 2021). Thus, the localization of NLRs is important and is likely regulated by proteins involved in trafficking (Figure 6).

### Regulation of NLR-signaling components

The correct localization of helper NLRs is likely important for signaling. For example, the helper NLR NRC4 accumulates at the extra-haustorial membrane following *P. infestans* infection (Duggan et al., 2021). In addition, the balanced activity of both cytosolic- and nuclear-EDS1 is required for full immunity (García et al., 2010). Thus, the localization of helper NLRs and NLR-signaling components is important for defense. The activity of NLR signaling components is also negatively regulated. The Arabidopsis RNL NRG1C functions as a negative regulator in NLR-mediated immunity; overexpressing *NRG1C* compromised TNL-mediated HR and resistance (Wu et al., 2021a). In addition, an atypical member of the NRC family, NRCX, negatively regulates other NRC members to modulate immunity (Adachi et al., 2021). Posttranslational modifications (PTMs) are important for the functions of both PRRs and NLRs. For example, the phosphorylation of the C-terminus of the TNL RRS1-R is crucial for its recognition of the effector PopP2 (Guo et al., 2020). It is currently unclear whether PTMs are important for the activation and/or stability of NLR-signaling components. Perhaps, EP-proteins and helper NLRs must also undergo PTMs in order to function properly. The additional regulation of NLR-signaling components pre-NLR activation and postNLR activation remains to be investigated (Figure 6).

## Suppression of immunity by effectors

Multiple effectors have been shown to target both the PRR- and NLR-signaling pathways. Here, we summarize our knowledge of effectors reported to target PTI or ETI. Unless specified, the effectors mentioned in this section are from various *P. syringae* strains. AvrPtoB is an E3 ubiquitin ligase that induces the degradation of FLS2 (Göhre et al., 2008; Lu et al., 2011). HopB1 specifically degrades activated BAK1 (Li et al., 2016). AvrPto targets SOBIR1 and the FLS2–BAK1 complex by inhibiting their kinase activities (Xing et al., 2007; Shan et al., 2008; Xiang et al., 2008; Meng and Zhang, 2013; Wu et al., 2017b). Similarly, the conserved *Colletotrichum* effector NIS1 also targets receptor kinase complexes (Irieda et al., 2019). The tyrosine phosphatase HopAO1 directly dephosphorylates EFR (Macho et al., 2014). As RLCKs are central immune regulators, they are targeted by multiple effectors. AvrAC from *X.* *campestris* uridylylates BIK1 and PBL2 (Feng et al., 2012; Wang et al., 2015). HopZ1a acetylates RLCKs, and AvrPphB is a cysteine protease that degrades RLCKs such as BIK1, PBS1, and PBL1 (Zhang et al., 2010a; Bastedo et al., 2019). Other downstream PRR signaling components are also targeted by effectors. The ADP-ribosyltransferase HopF2 targets both BAK1 and MKK5 to suppress PTI signaling (Wang et al., 2010; ZHou et al., 2014). HopAI1 inactivates MPK3, MPK4, and MPK6 via its phosphothreonine lyase activity (Zhang et al., 2007). AvrRpt2 suppresses MPK4/11 activation (Eschen-Lippold et al., 2016). Interestingly, many parallel mechanisms are employed to suppress the same PRR-signaling node in different hosts by different pathogens (Figure 6).

Phosphorylation of SGT1 by MAPKs is required for NLR activation, implying that NLRs are regulated by SGT1 following PTI-induced MAPK activation (Hoser et al., 2013; Yu et al., 2020). The *R.* *solanacearum* effector RipAC prevents MAPK-mediated phosphorylation of SGT1, which suppresses NLR-mediated immunity (Yu et al., 2020). Two effectors were recently shown to suppress NRC-mediated HR. The *P. infestans* effector AVRcap1b and the cyst nematode effector SPRYSEC15 can suppress autoimmunity induced by autoactive alleles of NRC2 and NRC3 (Derevnina et al., 2021). Suppression of NRC2 and NRC3 by AVRcap1b is dependent on the membrane trafficking-associated protein TARGET OF MYB 1-LIKE PROTEIN 9A (*Nb*TOL9a; Derevnina et al., 2021). AVRcap1b suppresses NRC2 and NRC3 by directly interacting with their NB-ARC domains (Derevnina et al., 2021). Another *Phytophthora* effector (from *Phytophthora* *capsici*), PcAvh103, suppresses immunity by promoting the disassociation of the EDS1–PAD4 complex (Li et al., 2020). More studies are needed to identify pathogen effectors that target the NLR signaling pathway.

In Arabidopsis, the transcription factors CALMODULIN-BINDING TRANSCRIPTION ACTIVATOR 1/2/3 (CAMTA1/2/3) and CBP60a negatively regulate defense-induced transcriptional reprogramming (Truman et al., 2013; Kim et al., 2020; Sun et al., 2020). Pathogens also target defense-related transcription factors to suppress immunity. For example, the *R.* *solanacearum* effector PopP2 acetylates and inhibits WRKY transcription factors to suppress immunity (Le Roux et al., 2015; Sarris et al., 2015; Zhang et al., 2017b). In addition, the *Verticillium dahliae* effector VdSCP41 inhibits SARD1 and CBP60g to facilitate its proliferation (Qin et al., 2018; Figure 6).

## The interactions between PTI and ETI

While PRR- and NLR-mediated immunity has been extensively studied for the last 20 years, it has not been clear how or if these defense mechanisms interact. NLR-mediated immunity is mostly activated in the presence of microbes or PAMPs. Most studies on NLR-mediated immunity have involved transient expression-based comparisons between PTI and “PTI + ETI.” The activation of NLRs in the absence of PTI has not been extensively studied until recently. There have been multiple reports on the different interactions between these two immune systems. Here, we describe three situations in which PTI and ETI interact with each other.

### NLRs guard the PRR-signaling pathway

**Figure 7:**
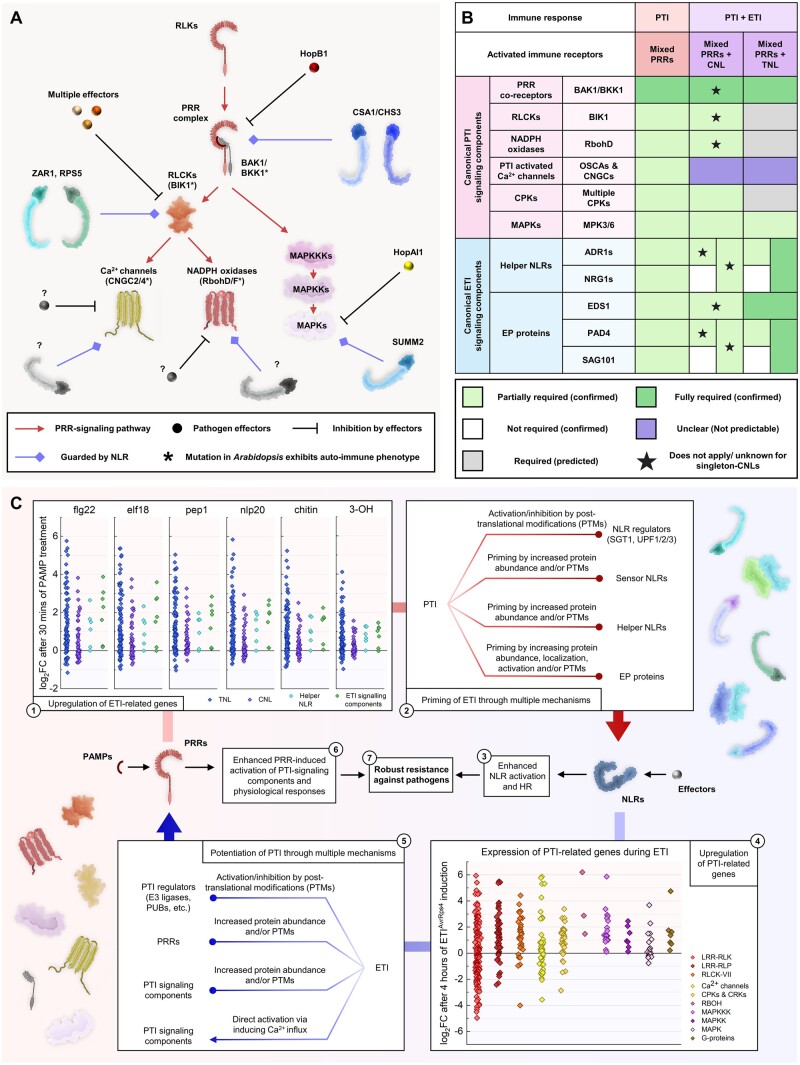
Interactions between PRR- and NLR-mediated immunity. A, NLRs guarding the PRR-signaling pathway. Multiple PRR-signaling components are suppressed by effectors. NLRs guard these signaling components and reverse susceptibility triggered by these effectors. Question marks indicate unidentified effectors or NLRs. B, Tabular summary of signaling components required for PRR- and NLR-mediated immunity. Green shading represents confirmed requirement from publications. Gray shading indicates predicted requirement. Purple shading represents unclear requirement that cannot be predicted. C, Mechanisms involved in the mutual potentiation between PRR- and NLR-mediated immunity. Transcriptomic data were obtained from previously published data (Bjornson et al., 2021; Ngou et al., 2021a). Numbers indicate the corresponding mechanisms to potentiate PRR- or NLR-mediated immunity to achieve robust resistance against pathogens.

Many effectors target the PRR-signaling pathway. Plants have evolved multiple NLRs to detect these effectors via the guarding of PRR-signaling components or decoys. As a result, many PRRs and PRR-signaling loss-of-function mutants, such as the Arabidopsis mutants *bak1-4 bkk1-1*, *bik1*, *cngc2/4*, *rbohd/f*, *mekk1*, *mkk1/2*, *mpk4*, and *camta3*, exhibit autoimmune phenotypes (Torres et al., 2002; Roux et al., 2011; Zhang et al., 2012; Chen et al., 2016; Liu et al., 2017; Lolle et al., 2017; Kadota et al., 2019; Tian et al., 2019). The autoimmunity observed in some of these mutants is caused by the activation of multiple NLRs. The TNL CONSTITUTIVE SHADE-AVOIDANCE 1 guards both BIR3 and BAK1 (Schulze et al., 2021). In addition, *bak1-3 bkk-1-*autoimmunity and HopB1-triggered immunity are dependent on ADR1s (Wu et al., 2020b). RLCKs are targeted by multiple effectors. The CNL ZAR1 together with the RLCK RKS1 monitor PBL2, and the CNL RPS5 monitors PBS1, to reverse ETS (Shao et al., 2003; Zhang et al., 2010a; Wang et al., 2015). The CNL SUMM2 guards and senses the disruption of the MEKK1–MKK1/2–MPK4 kinase cascade via CALMODULIN-BINDING RECEPTOR-LIKE CYTOPLASMIC KINASE 3, a substrate protein of MPK4 (Zhang et al., 2012, 2017a). SUMM2 also detects the *P. syringae* effector HopAI1, which inhibits MPK4 kinase activity (Zhang et al., 2012). The TNL RPS6 also contributes to HopAI1-triggered immunity (Takagi et al., 2019). Whether the autoimmunity in *bik1*, *cgnc2/4*, and *rbohd/f* is dependent on NLRs remains unclear. Other NLRs that guard the PRR-signaling pathway remain to be identified (Figure 7A).

### Interdependency of signaling components between PRRs and NLRs

PRR co-receptors, RLCKs, NADPH oxidases, calcium channels, CPKs, and MAPKs are considered to be canonical PRR-signaling components, while EP proteins and helper NLRs are considered to be canonical NLR-signaling components. However, recent studies indicated that PRR-mediated resistance is dependent on canonical NLR-signaling components and vice versa (Ngou et al., 2021a; Pruitt et al., 2021; Tian et al., 2021; Yuan et al., 2021; Figure 7B). As mentioned, flg22- and nlp20-induced resistance is partially dependent on EDS1, PAD4, SAG101, ADR1s, and NRG1s (Pruitt et al., 2021; Tian et al., 2021). Pruitt et al. (2021) proposed that EP-proteins and helper NLRs are activated by RLPs through interactions between RLP co-receptors (SOBIR1), EP-proteins, and helper NLRs, although it remains to be determined whether EP-proteins play a primary or secondary role in RLP defense signaling. Another report, however, suggested that the activation of PRRs leads to increased expression of multiple NLRs and other TIR-domain-containing proteins, promoting downstream signaling (Tian et al., 2021). These two hypotheses are not mutually exclusive, and the exact mechanisms by which PRR-mediated immunity involves NLR-signaling components remain to be determined.

NLR-mediated immunity is also dependent on PRRs and multiple PRR-signaling components. In Arabidopsis, RPS2-, RPS5-, and RRS1/RPS4-mediated resistance is dependent on BAK1 and BKK1 (Ngou et al., 2021a; Yuan et al., 2021). RPS2-mediated resistance is also dependent on BIK1 and RbohD (Kadota et al., 2019; Yuan et al., 2021). Both RPM1- and RPS2-mediated resistance and the HR are dependent on CPK1/2/5/6 (Gao et al., 2013). The activation of MPK3 and MPK6 is also required for the HR and resistance mediated by multiple NLRs including RPM1, RPS2, RPS5, and RRS1/RPS4 (Su et al., 2018). One of the proposed key mechanisms by which ETI halts pathogen infection is to potentiate and restore PTI from turnover and the action of pathogen effectors (Ngou et al., 2021a; Yuan et al., 2021). As a result, PRRs and PRR-signaling components are required for NLR-mediated resistance. The molecular mechanisms by which ETI potentiates PTI will be discussed in the next section.

### Mutual potentiation between PRR- and NLR-mediated immunity

Activation of the TNLs RRS1/RPS4 and RPP4 using an estradiol-inducible recognized effector (ETI without PTI) did not trigger the HR. The presence of PAMPs/MAMPs restored the HR induced by these TNLs (Ngou et al., 2020, 2021a). Similarly, the HR induced by the CNLs RPM1, RPS2, and RPS5 was also potentiated by the activation of PRRs (Ngou et al., 2021a). In addition, the HR and resistance induced by RPS2 are compromised in PRR mutants (Ma et al., 2012; Yuan et al., 2021). There are a few possible mechanisms by which PRRs potentiate NLR-induced immunity. First, the activation of PRRs could induce the expression of NLRs and NLR-signaling components (Navarro et al., 2004; Bonardi et al., 2011; Brendolise et al., 2018; Jung et al., 2020). A recent transcriptomics study suggested that the activation of different PRRs induces highly overlapping transcriptional changes (Bjornson et al., 2021). Indeed, the activation of six distinct PRRs led to the upregulation of genes encoding most TNLs, CNLs, EP-proteins, and helper NLRs in Arabidopsis (Bjornson et al., 2021; Figure 7C;  Supplemental Data Set 3). The increased abundance of these proteins might therefore “prime” the activation of NLRs upon effector recognition. Second, the activation of PRRs might prime NLR-mediated immunity via PTMs. Upon PAMP perception, SGT1 is phosphorylated by MAPKs, which is important for the stability of NLRs (Yu et al., 2020). In addition, nonsense-mediated decay of *NLR* transcripts is inhibited upon PAMP recognition (Jung et al., 2020). Thus, the stability of NLRs can be affected by both transcriptional and posttranscriptional modifications activated by PTI. Conceivably, EP proteins and helper NLRs might also be primed via PTMs induced by PTI. Flg22 treatment led to reduced polyubiquitination levels of EDS1 (Grubb et al., 2021; Ma et al., 2021). Whether and how PTI primes NLR-signaling components remain to be investigated.

The activation of NLRs potentiates PAMP-induced cellular responses, such as ROS production, callose deposition, and defense-related gene expression (Ngou et al., 2021a). The activation of multiple PRR signaling components, such as BIK1, RbohD, and MPK3, is also potentiated by ETI (Ngou et al., 2021a; Yuan et al., 2021). ETI induces the transcript and protein accumulation of SOBIR1, BAK1, BIK1, RbohD, and MPK3 (Ngou et al., 2021a). Transcriptomic analysis confirmed that multiple PRR signaling components are also upregulated upon the activation of RRS1/RPS4. These include CPK1/2/5/6, XLG2, and the calcium channels OSCA1.3, CNGC19/20, GLR2.7/2.8/2.9 (Ngou et al., 2021a; Figure 7C;  Supplemental Data Set 4). Interestingly, the transcript levels of *BIK1*, *MPK3*, and *RbohD* are only transiently upregulated during ETI. However, the protein levels of these genes remain upregulated for an extensive period of time (Ngou et al., 2021a). This implies that PTMs or other posttranscriptional mechanisms might also influence the stability of PRR-signaling components during ETI. The protein abundance of PRR signaling components, such as BAK1, BIK1, and RbohD, is tightly regulated by multiple processes (Figure 6). How ETI regulates or affects these processes remains unclear. In addition, calcium influx induced by NLRs might contribute to the potentiation of PTI through CPKs (Bi et al., 2021; Jacob et al., 2021; Ngou et al., 2021b). To summarize, PTI and ETI mutually potentiate each other through multiple mechanisms to induce robust immunity against pathogens (Figure 7C).

## Historic overview of research in PTI and future challenges

**Figure 8:**
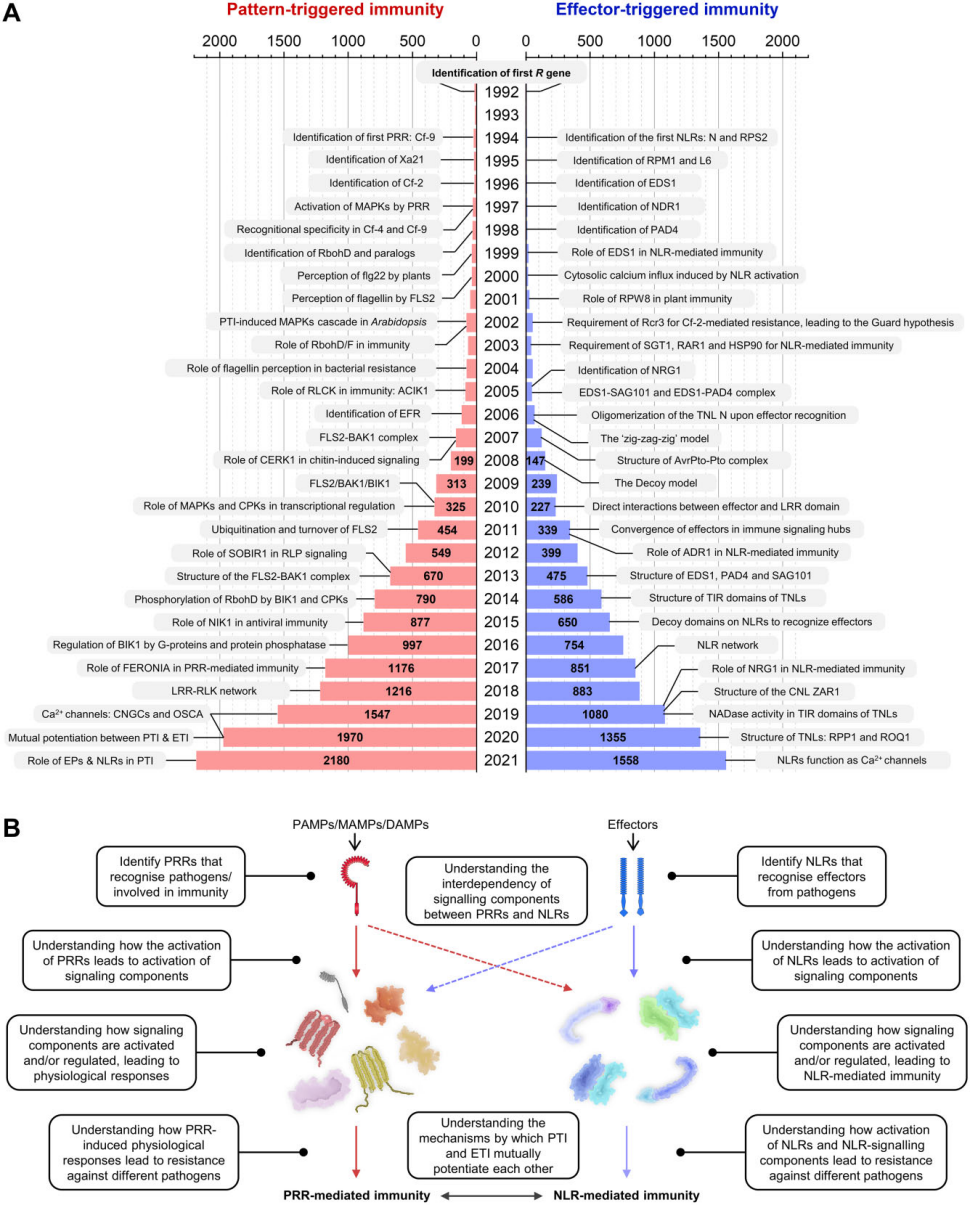
Historic overview of PTI and ETI and future challenges. A, Discoveries in PTI (left) and ETI (right) in the past 30 years. Bar charts represent the number of “plant biology” publications that mentioned “pattern-trigger immunity” (red) and “effector-triggered immunity” (blue). Data obtained from Dimensions (https://www.dimensions.ai/). B, Future challenges and outlook in plant immunity research.

Researchers identified the first PRR-encoding gene, *Cf-9*, back in 1994 (Jones et al., 1994). Multiple PRR genes, such as *Xa21*, *Cf-2*, *Cf-4*, *FLS2*, *EFR*, and *RLP23*, were subsequently identified and used as models to study PTI (Song et al., 1995; Dixon et al., 1996; Thomas et al., 1997; Gómez-Gómez and Boller, 2000; Zipfel et al., 2006). Researchers then explored PRR-induced physiological responses and identified multiple signaling components. The activation of MAPKs by cell-surface receptors were reported back in 1997 (Ligterink et al., 1997) and was verified for Cf- genes 2 years later (Romeis et al., 1999). In tobacco (*N.* *tabacum*), the perception of PAMPs leads to the activation of wounding-induced protein kinase (WIPK) and SA-induced protein kinase (SIPK; Zhang and Klessig, 1998; Yang et al., 2001). WIPKs and SIPKs are orthologs of the subsequently identified Arabidopsis MPK3 and MPK6, respectively (Asai et al., 2002). Accumulation of ROS and callose deposition during infection were also reported in 1997 (Thordal-Christensen et al., 1997), and for Cf-initiated responses (Piedras et al., 1998). Researchers identified the human Rbohs in Arabidopsis and showed that two of these (RbohD and RbohF) are required for ROS production during infection (Torres et al., 1998, 2002). It was unclear how these signaling components were activated by PRRs until the identification of the PRR co-receptors and RLCKs. BAK1 was identified as a co-receptor essential for FLS2-mediated resistance in 2007 (Chinchilla et al., 2007). In the same year, CERK1 was also shown to be essential for chitin-mediated immunity (Miya et al., 2007). In 2013, SOBIR1 was identified as a co-receptor of RLPs, and the structure of the FLS2/BAK1 receptor complex was also defined (Liebrand et al., 2013; Sun et al., 2013). In 2018, a genome-wide analysis of Arabidopsis LRR–RLKs interactions was reported, further supporting the theory that PRRs interact with each other to modulate and transduce signals (Smakowska-Luzan et al., 2018). Tomato ACIK1 was the first RLCK shown to be an essential signaling component in PRR-mediated immunity (Rowland et al., 2005). The Arabidopsis ortholog BIK1 was subsequently shown to be a central PRR-signaling component (Lu et al., 2010; Zhang et al., 2010a). RbohD, MAPKKKs, and multiple calcium channels were shown to be phosphorylated by RLCKs, which leads to downstream immune responses (Boudsocq et al., 2010; Kadota et al., 2014; Li et al., 2014; Yamada et al., 2016; Bi et al., 2018; Tian et al., 2019; Thor et al., 2020; Figure 8A).

More than 60 immunity-related PRRs with known ligands have now been identified. Arabidopsis EFR has been introduced into multiple plant species, such as tomato, rice, orange, and apple, providing broadspectrum resistance to many bacteria (Lacombe et al., 2010; Schwessinger et al., 2015; Mitre et al., 2021; Piazza et al., 2021). Therefore, the identification of novel PRRs that recognize PAMPs or other elicitors would provide resources to engineer disease-resistant crops. Other challenges in PRR biology include trying to understand how PRRs activate downstream signaling components and physiological responses, how these processes are regulated and suppressed by effectors, and how resistance against pathogens is achieved (Figure 8B).

## Historic overview of research in ETI and future challenges

Arabidopsis *RPS2* and the tobacco *N* gene were the first reported NLR genes (Bent et al., 1994; Mindrinos et al., 1994; Whitham et al., 1994). Multiple NLRs, including RPM1 and L6, were subsequently identified (Grant et al., 1995; Lawrence et al., 1995). Understanding how NLRs detect effectors has led to multiple models. The guard hypothesis was proposed to explain how the protein kinase Pto confers Prf-dependent recognition of AvrPto (Van der Biezen and Jones, 1998). Many other examples have emerged that are consistent with this hypothesis, such as the requirement of the protease Rcr3 for Cf-2-mediated resistance (Van der Biezen and Jones, 1998; Dangl and Jones, 2001; Krüger et al., 2002). The decoy model was then proposed, which is further supported by the discovery of integrated decoy domains in NLRs (van der Hoorn and Kamoun, 2008; Cesari et al., 2014; Le Roux et al., 2015; Sarris et al., 2015, 2016). The discovery of NRCs led to the concept of NLR networks (Gabriëls et al., 2007; Wu et al., 2017a, 2018). Following the identification of multiple NLRs, researchers identified multiple genetic components required for NLR-mediated immunity. These include EDS1, NDR1, PAD4, RPW8, SGT1, RAR1, HSP90, SAG101, NRG1s, and ADR1s (Parker et al., 1996; Century et al., 1997; Zhou et al., 1998; Falk et al., 1999; Xiao et al., 2001; Azevedo et al., 2002; Takahashi et al., 2003; Feys et al., 2005; Peart et al., 2005; Bonardi et al., 2011). EDS1 was later shown to co-function with SAG101 and PAD4 to mediate HR and resistance during ETI (Feys et al., 2001, 2005; Wagner et al., 2013; Sun et al., 2021; Wu et al., 2021b). Similarly, ADR1 and NRG1 have been shown to function downstream of multiple sensor NLRs to mediate the HR and resistance (Castel et al., 2019a; Wu et al., 2019; Saile et al., 2020). How sensor NLRs activate these signaling components is currently under investigation. v-cADPR produced by TIR domains might contribute to the activation of EP-proteins and helper NLRs (Horsefield et al., 2019; Wan et al., 2019a, 2019b). NLRs were shown to oligomerize and trigger cytosolic calcium influx following effector recognition (Grant et al., 2000; Mestre and Baulcombe, 2006). The discovery of the structures of multiple NLR resistosomes proved that the oligomerization of NLRs is required for resistance, likely through the formation of cation channels (Wang et al., 2019a; Ma et al., 2020a; Martin et al., 2020; Bi et al., 2021; Jacob et al., 2021). However, oligomerization of TIR domains imposed by an NLRC4 scaffold is sufficient to activate defense (Duxbury et al., 2020; Figure 8A).

More than 140 NLRs with known recognized effectors have been identified (Kourelis and Kamoun, 2020). Cross-species transfer of NLR “stacks” provides durable resistance against pathogens (Jones et al., 2003; Mukhtar, 2013; Ghislain et al., 2019; Luo et al., 2021; Witek et al., 2021). Identification of novel NLRs will provide resources to engineer crop resistance against multiple pathogens. Current challenges in NLR biology include understanding how NLRs activate downstream signaling components, how these signaling components then trigger immune responses, how these processes are regulated and suppressed by effectors, and how NLRs and PRRs co-function to achieve resistance against pathogens (Figure 8B).

## Conclusion and perspectives

Plants respond to pathogens using a two-tier innate immune system activated by both cell-surface and intracellular immune receptors. The perception of PAMPs/MAMPs/DAMPs/HAMPs on the cell surface leads to PRR-mediated immunity, and the recognition of effectors leads to intracellular NLR-mediated immunity. The first plant *Resistance* (*R*) gene, *Hm1*, was cloned back in 1992 (Johal and Briggs, 1992). Many immune receptors have been identified since 1994, when the first PRR and NLRs were identified. Tremendous efforts have been made to understand the PRR- and NLR-signaling pathways. PRRs and NLRs utilize some overlapping but also unique signaling components to activate each of their downstream physiological responses, which thwart pathogen proliferation. Both signaling pathways are tightly regulated to prevent autoimmunity, while being suppressed by pathogen effectors. Recent studies have shown that PRR- and NLR-mediated immunity can be mutually potentiated and are dependent on each other. Great opportunities for novel discoveries remain in addressing the following challenges in the research of plant immunity: (1) identifying novel immune receptors; (2) understanding the signaling pathways and physiological responses triggered by both cell-surface and intracellular immune receptors; (3) understanding how immunity is intrinsically regulated and manipulated by external biotic and/or abiotic factors; (4) understanding the vastly diverse mechanisms by which plants resist pathogen infections; and (5) understanding how different immune systems function synergistically during infections. These challenges overlap with some of the “top 10 unanswered questions in molecular plant-microbe interactions” (Harris et al., 2020) and will shape our understanding of plant immunity in the coming decades (Figure 8B).

## Supplemental data

The following materials are available in the online version of this article.

**Supplemental Data Set 1.** PRRs involved in plant immunity.

**Supplemental Data Set 2.** NLRs involved in plant immunity.

**Supplemental Data Set 3.** Expression of ETI-related genes during PTI.

**Supplemental Data Set 4.** Expression of PTI-related genes during ETI.

## Supplementary Material

koac041_supplementary_data

## Notes

B.P.M.N., P.D., and J.D.G.J. conceptualized the review outline. B.P.M.N. curated information, prepared the figures, and wrote the original draft, and B.P.M.N., P.D., and J.D.G.J. reviewed and edited the manuscript.

The author responsible for distribution of materials integral to the findings presented in this article in accordance with the policy described in the Instructions for Authors (https://academic.oup.com/plcell) is: Bruno Pok Man Ngou (pokman.ngou@riken.jp).
